# Vortex-Based Cavitation Devices for Continuous Emulsification: Influence of the Device Design, Scale-Up, and Scale-Out

**DOI:** 10.1021/acs.iecr.6c00278

**Published:** 2026-04-08

**Authors:** Amol Gode, Kyriakos Kourousis, Vivek V. Ranade

**Affiliations:** † Multiphase Reactors and Intensification Group Bernal Institute, 8808 University of Limerick, Limerick V94T9PX, Ireland; ‡ School of Engineering, University of Limerick, Limerick V94T9PX, Ireland

## Abstract

Continuous emulsification is a desirable alternative to batch processing, especially in sectors such as food and beverage, cosmetics, and pharmaceuticals, where large volumes of consistent emulsions are required. Vortex-based hydrodynamic cavitation (VD) devices have emerged as efficient and scalable options using cavitation-induced local energy dissipation to promote droplet breakup. However, practical guidance for device design and pathways for scale-up and scale-out remains limited. This study investigates the influence of the outlet configuration, chamber geometry, number of inlets, and scale-up or scale-out on the performance of VD for continuous production of liquid–liquid emulsions. The results indicate that including a vortex stabilizer in the chamber and using a multiple-inlet device improved the cavitation activity. Geometrically similar scale-up (1–5 LPM) resulted in a slight increase in the Sauter mean diameter and lower energy efficiency, which confirmed the flow characteristics related to the distribution of turbulence dissipation rates becoming attenuated at larger scales. The scale-out options were investigated considering two and four 1 LPM nominal flow rate devices operated in parallel to increase the nominal flow rate to 5 LPM for the four devices. The results confirm that while scale-up and scale-out have limited impact on final droplet size distribution, they influence energy effectiveness. Multi-inlet designs were seen to enhance emulsification efficiency and reduce droplet size, especially when operating at low pressure drop values. Overall, this study provides practical guidelines for designing and deploying vortex-based HC devices for continuous emulsification applications.

## Graphical abstract

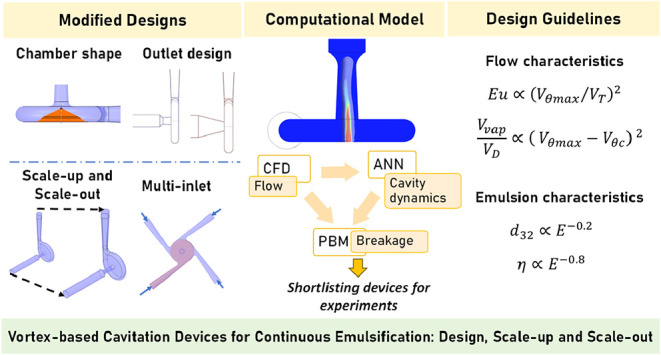

## 1 Introduction

Emulsions are a critical part of many industries, including food and beverage, pharmaceuticals, and chemicals. For such sectors, the continuous production of emulsions is increasingly important because large and consistent volumes must be manufactured with reliable quality. Compared with batch operation, continuous emulsification offers several advantages, including higher throughput, improved consistency, simplified scaling, and a smaller footprint. Continuous emulsification is achieved by continuously feeding aqueous and organic phases into an emulsification device, where high shear forces break the dispersed phase into fine droplets, and the resulting emulsion is continuously discharged. Conventional emulsification equipment includes high-pressure homogenizers, rotor stators, and ultrasonic systems.^1–5^ While high-pressure homogenization (HPH) and microfluidization excel at producing nanoscale droplets (<500 nm), the production of micron-sized emulsions (1–10 μm) remains a critical requirement across several industries. Macroemulsions are relevant in the following industries:

- Food and beverage industry: Droplet size significantly dictates sensory perception. Emulsions in the 1–10 μm range are essential for providing “creaminess,” “opacity,” and specific mouthfeel in dairy alternatives, sauces, and dressings. Nanoemulsions often fail to provide the desired textural properties and can lead to a “watery” perception.^6,7^

- Pharmaceuticals and controlled release: For certain drug delivery systems, larger droplets at 1–10 μm are required to control the release kinetics of encapsulated bioactive compounds. Micron-sized droplets can provide a more sustained release profile compared to the rapid burst release typically associated with nanoemulsions.^8^
- Agrochemicals: In pesticide and herbicide formulations, larger droplets are often preferred to enhance adhesion to leaf surfaces and minimize “spray drift,” a significant environmental concern associated with finer droplets.^9^

Hydrodynamic cavitation (HC) has emerged as a highly promising and energy-efficient technology for process intensification across a range of applications, including wastewater treatment, crystallization, and liquid–liquid emulsification.^10^ In recent times, hydrodynamic cavitation devices for emulsion production are gaining impetus.^11,12^ In HC systems, emulsification occurs through a combination of turbulent shear and cavitation mechanisms. The controlled formation, growth, and violent collapse of vapor cavities in a flowing liquid generate intense local shear and shock waves that efficiently fragment large droplets. Key advantages of HC include its scalability and compatibility with continuous operation. In this work, we focus on HC-induced continuous emulsification. The use of linear flow devices for emulsion production is mostly accomplished with devices with constriction, including orifices and venturis. On the other hand, the swirling flow devices studied for emulsification include the vortex-based devices, swirling jet devices, and venturis with a swirler.

VDs offer an attractive platform for emulsification^13–17^ with several operational advantages over orifice-based HC, HPH, or microfluidizers for producing macroemulsions. Some of these are outlined as follows.

- Less susceptibility to clogging and erosion: HPH and microfluidizers rely on extremely narrow interaction chambers or gaps (10–100 μm), which make them susceptible to clogging and erosion. Unlike these, VDs feature a large, open clearance chamber and mm-scale smaller dimensions, making it uniquely robust for processing complex industrial fluids with orders of magnitude lower erosion and clogging than other competing devices.
- Scalability and maintenance: VDs have no moving parts and operates at significantly lower pressure drops (∼10° bar). This results in significantly lower CAPEX and OPEX compared to other competing high-pressure systems (operating pressure drops > ∼10^1^ bar). As demonstrated in our comparison between VD3 and VD6, the VD offers predictable performance during scale-up, making it a reliable solution for high-throughput continuous manufacturing.
- Stability: Emulsions produced through VDs exhibit excellent stability, and DSD remains stable for more than 60 days.^18^

The VD is positioned not as a direct competitor to nanohomogenizers but as a robust, industrial-scale alternative for applications where nanoscale is neither necessary nor desirable. The VD creates a swirling flow to create a low-pressure vortex core, inducing cavitation at lower inlet pressures and away from the device walls, thereby reducing erosion risks. The implosion of cavities generates highly localized, intense shear and high energy dissipation rates, which interact with the dispersed phase to generate fine emulsions. Thus, using VDs, we obtain high-quality emulsions at low operating pressure drops and low costs. Our research group has recently demonstrated that VDs are highly effective in producing liquid–liquid emulsions,^13–19^ showing versatility across various oil and aqueous phase systems, including dense rapeseed oil-in-water^16^ and coconut oil-in-water^15^ emulsions. In the present work, we leverage these benefits for the continuous generation of rapeseed oil-in-water emulsions using different configurations of the VD. The state-of-the-art use of VDs for emulsification is briefly reviewed in the following.

The internal flow characteristics of VDs govern the effectiveness of VDs for end applications. This has been extensively studied by many researchers including Priestman,^20,21^ Vatistas et al.,^22^ Kulkarni et al.,^23,24^ Pandare and Ranade,^25^ Simpson and Ranade,^26^ and, recently, Gode et al.^27^ These investigations established the importance of geometric parameters such as the chamber aspect ratio, scale, and number of tangential inlets on the hydrodynamic performance of the VD (e.g., Euler number) and the extent of cavitation (generated vapor volume). Gode et al.^27^ investigated the influence of the aspect ratio, scale, and number of inlets on the hydrodynamic performance. The computational results suggested that geometric scale-up methods had an adverse effect on performance, recommending scale-out as a potential alternative. However, these flow studies, including initial investigations by Gode et al.^27^ on the influence of the number of tangential inlets, were restricted to constant flow conditions and lacked validation against an end application (for example, emulsion production).

The application of VDs for continuous emulsification has also seen a targeted development. Experimentally, Gode et al.^17^ compared the performance of different scales of VDs with some commercial devices and inferred that the lab-scale VD was superior for continuous emulsion production, supporting the viability of a scale-out approach using lab-scale units. For realizing optimum design and operating parameters for continuous emulsions with a desired DSD, modeling of the process becomes essential. The modeling of continuous emulsification with VDs was recently investigated by Gode and Ranade,^28^ who introduced a novel ensemble approach (CFD+PBM+ANN). This multiscale approach incorporates the macroscale study of flow behavior in the VD through CFD simulation, estimation of the localized energy dissipation rate in the effective cavitation region using an ANN-based surrogate model (microscale), and finally DSD prediction using PBM simulation. This modeling framework provides the required means for simulating droplet breakage in continuous emulsification with VDs. Based on the above discussion, the key gaps in the current state of the art include (a) the influence of complex geometric modifications (e.g., outlet design, chamber shape) on flow and emulsion characteristics in a continuous setup and (b) the emulsification performance of VDs at varying throughput accomplished by either scale-up (geometric similarity), increasing the number of inlets (more flow to achieve the same pressure drop), or scale-out (connecting multiple VDs in parallel). The present study aims to bridge these gaps by investigating the influence of the outlet configuration, vortex chamber design, scale-up, number of tangential inlets, and scale-out on flow (Euler number, swirl ratio, generated vapor, and localized energy dissipation rates via cavity collapse) and emulsification performance (DSD, Sauter mean diameter, *d*_32_, and energy efficiency of emulsification, η). The objective is to identify device designs and operating strategies that deliver the desired emulsion characteristics and production throughput, while maintaining favorable energy efficiency. The first part of the study computationally investigates the influence of key geometric modifications and their influence on flow characteristics and emulsification performance. The shortlisted designs are then fabricated using stereolithography (SLA)-based additive manufacturing and experimentally validated. DSD, *d*_32_, and η are analyzed. The results provide essential design guidelines for continuous emulsification by using VDs.

## 2 Exploring Design Space and Shortlisting for Experiments

The primary motivation for these specific modifications is to investigate the VD for improvements in performance, capacity, and energy efficiency for continuous emulsification. The modifications and the design parameters investigated along with the naming convention for the vortex-based HC devices are listed in Table 1.

**Table 1:** List of Modifications to Vortex-Based HC Devices

device names	throat diameter (*d*_T_), mm	chamber shape, -	outlet port modifications, -	number of inlets, -	number of parallel devices, -
VD3	3	cylindrical	standard	1	1
VD6	6	cylindrical	standard	1	1
VD3_MO1	3	cylindrical	ER_o_ = 2.5	1	1
VD3_MO2	3	cylindrical	*l*_o_ = 4.38 mm	1	1
VD3_MC1	3	vortex stabilizer	standard	1	1
VD3_MC2	4.35	volute shape	ER_o_ = 1	1	1
VD3_2N	3	cylindrical	standard	1	2
VD3_4N	3	cylindrical	standard	1	4
VD3_2I	3	cylindrical	standard	2	1
VD3_4I	3	cylindrical	standard	4	1

The influence of variation in geometric design parameters of the VD on the flow performance was studied by several researchers; Kulkarni et al.^23,24^ provided design guidelines based on diodicity; Simpson and Ranade^26^ studied the effect of scale, and Gode et al.^27^ investigated the influence of the aspect ratio, scale, and number of inlets on the flow performance of the VD. In the study by Gode et al.,^27^ although the aspect ratio and scale were varied, a complex variation in the internal shape of the chamber was not considered and the influence of the number of inlets was evaluated at the same flow rate and pressure drop condition. In this study, modifications to the chamber shape and outlet ports are considered to further improve the internal flow dynamics and cavitation extent with respect to the standard VD design. The outlet port geometry directly influences the axial length of the low-pressure vortex core. Modifying the expansion ratio and length is targeted toward controlling the cavitation zone and maximizing the probability of oil droplets encountering this cavitation region. The chamber shape modification by including a vortex stabilizer in the chamber is aimed toward stabilizing the swirling flow, maintaining it at the axial position and potentially reducing vortex precession as well as the pressure drop. The number of tangential inlets (see the ‘Number of inlets’ column in Table 1) defines the swirl intensity and symmetry of the vortex core. Increasing the number of inlets (from the base case 1 to 2 and 4) would lead to a symmetrical vortex and improved throughput, while retaining the characteristics of the single inlet design. The impact of geometric scale-up is obtained by comparing the performance of a lab-scale (VD3) device with an intermediate-scale (VD6) one. The feasibility of scale-out needs to be checked as an alternative to scale-up as the base design (VD3) was reported to give the best performance.^17^ In this context, the “Number of devices” column in Table 1 refers to the number of VD units (each unit having a single inlet) operated in a parallel configuration to increase the flow rate through the system at the same operating pressure drop. The scale-out is achieved by operating the VD3 in parallel with two and four devices. This study with two and four parallel standard VD devices will provide information on whether scale-out (which maintains the flow dynamics of the smaller VD) is a better strategy for achieving high throughput, while maintaining the similar DSD and energy efficiency of the lab-scale VD. In summary, three modifications to the standard VD are investigated: geometric optimization (chamber shape, outlet port design), scale-up performance (VD6), and scale-out feasibility (multiple inlets or multiple efficient small units operated in parallel to deliver high throughput at consistent quality). The different designs simulated in this work are shown in Figure 1. The simulation results were used to shortlist the designs for further investigations through experiments.

**Figure 1:**
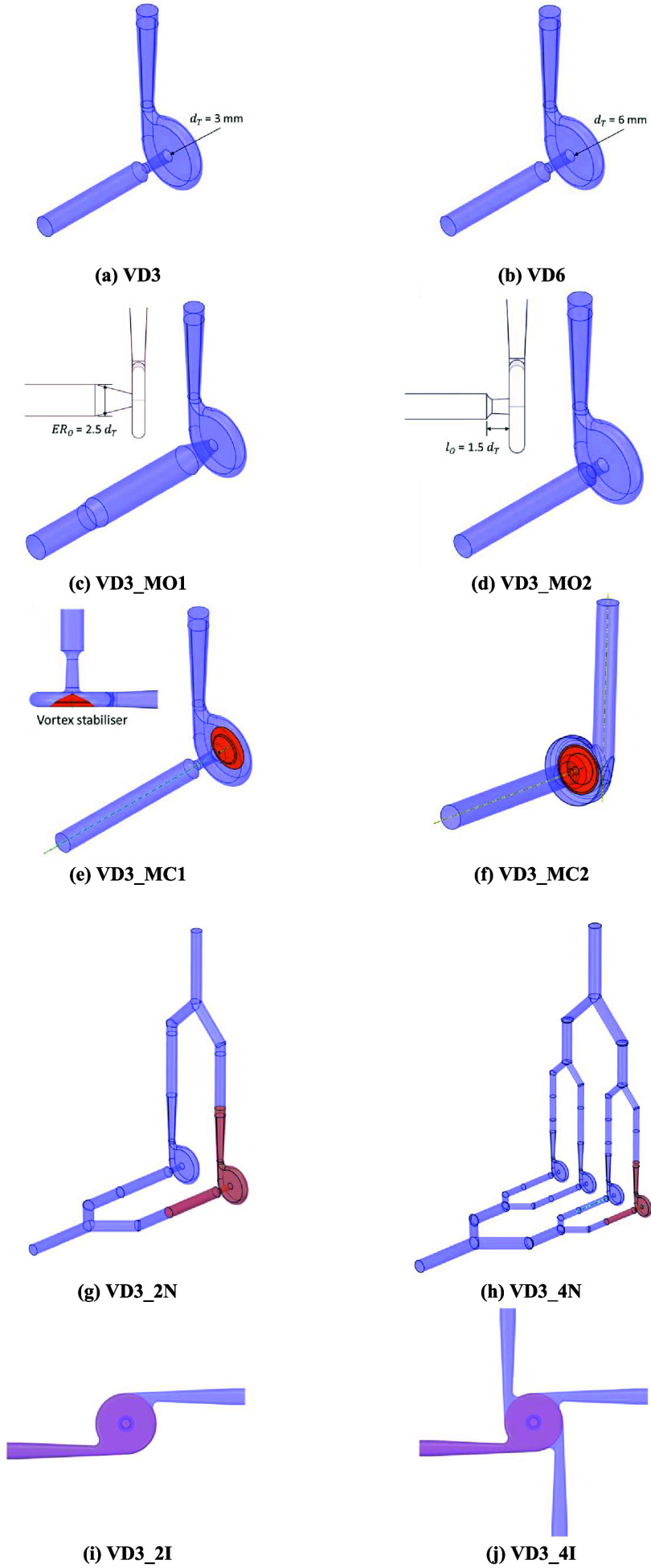
Vortex-based cavitation devices considered in this study.

### 2.1 Computational Model

The computational study was performed to investigate the flow and emulsification characteristics of the ten distinct VD configurations and provides an opportunity to shortlist the devices before going for actual fabrication and experimental evaluation. The influence of individual geometric modifications, geometric scale-up via increased size, and scale-out via parallelization on the resulting flow and emulsion characteristics was studied. The device configurations as given in Table 1 were included for the CFD+PBM simulations. The ensemble approach integrating CFD, ANN, and PBM, based on the work of Gode and Ranade,^28^ was employed for comparing the flow features and predicting the DSD of emulsions produced continuously using different VD modifications. This strategy addresses the challenge of accurately quantifying the highly localized and instantaneous energy dissipation generated by cavitation. The workflow proceeds in three sequential steps: CFD → ANN → PBM.

The swirling flow and cavitation within the VD were simulated using unsteady Reynolds-averaged Navier–Stokes (URANS) equations. The Eulerian mixture model was selected to represent the three-phase flow, continuous water phase (ρ_a_ = 1000 kg/m^3^ and μ_a_ = 0.001 Pa·s), dispersed oil phase (ρ_o_ = 915 kg/m^3^ and μ_o_ = 6.0 × 10^–2^ Pa·s), and water vapor phase (ρ_a_ = 0.55 kg/m^3^ and μ_a_ = 1.34 × 10^–5^ Pa·s). The shear stress transport (SST) k-ω turbulence model was utilized to obtain the shear-induced turbulent energy dissipation rate (ε) in the computational domain. Cavitation was modeled using the full cavitation model of Singhal^29^ as it accounts for the effects of noncondensable gases and the turbulent kinetic energy on the vaporization and condensation rates. Additional details on the model equations are included in Section S1 (Supporting Information, SI). The CFD solution is used to identify the effective cavitation region where the probability of droplet breakage due to cavitation is the highest. The discussion on the selection of specific modeling approaches is discussed by Gode and Ranade^28^ and is not repeated here for brevity.

The computational domain included only the emulsification device with inlet/outlet ports and a portion of the circulation loop. The circulation loop was modeled by appropriate boundary conditions as discussed by Gode and Ranade.^28^ A schematic of the solution domain for replicating a continuous emulsification process is shown in Figure 2.

**Figure 2:**
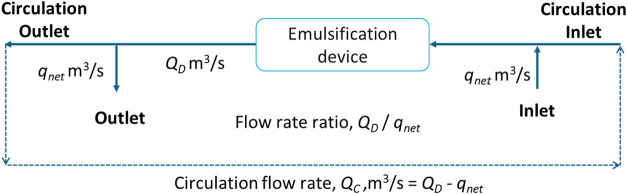
Schematic of the simulation domain for a continuous process (dotted line showing the circulation loop is not included in the computational domain).

All of the simulations were executed using commercial CFD software ANSYS Fluent (version 2024 R1). The governing URANS and multiphase equations were solved iteratively by using a segregated solver approach. The SIMPLE (semi-implicit method for pressure-linked equations) algorithm was employed for pressure–velocity coupling. To maintain high numerical accuracy, the second-order upwind schemes were utilized for the spatial discretization of momentum, turbulence kinetic energy (*k*), and specific dissipation rate (ω). The solution was deemed converged when the scaled residuals for all primary governing equations fell below 10^–3^ and the monitored integral parameters, specifically the domain-averaged mass flow rate and the pressure drop across the device, were stable.

The major difference in the simulation model adopted in this study pertains to the definition of the effective cavitation region. In the earlier work,^28^ the upper (α_vu_) and lower (α_vc_) limits of vapor fraction determining the cavitation zone were set to 0.6 and 0.1, respectively. The upper (α_vu_) and lower (α_vc_) limits of vapor fraction determine the cavitation zone and therefore have a physical significance. The upper limit was set to 0.6, which is close to the maximum packing vapor fraction, beyond which the gas phase may be treated as a continuous phase and there will not be any cavity collapse. The lower limit (α_vc_) defines the overall size of the cavitation zone as shown in Figure 3a. In this work, the entire cavitation zone was represented by one value of the energy dissipation rate induced by cavity collapse (ε_cav_). In that sense, α_vc_ may be treated as a model parameter with a specific hydrodynamic significance. In our earlier work,^28^ where experimental data were obtained at pressure drops much higher than the inception pressure drop (close to 50 kPa), the lower limit (α_vc_) was set to 0.1. In this work, experiments were also carried out very close to cavitation inception (at a pressure drop across the VD as 50 kPa). These experiments showed ∼1 mm droplets in the measured DSD, which will not otherwise form in the absence of cavitation. The setting of the lower limit (α_vc_) to 0.1 was not able to adequately capture the droplet breakage seen in the experiments conducted at 50 kPa. Lowering the α_vc_ to 0.02 expanded the modeled cavitation region (as shown in Figure 3c) and resulted in better agreement between the simulated and experimental DSD obtained in the 50 kPa case. Once the lower limit (α_vc_) was set to 0.02 based on the experimental data obtained at 50 kPa with VD3, it was kept the same for all other operating conditions and devices without any further adjustment. This reduction in the lower limit enlarged the cavitation zone, as seen from the effective cavitation zones for VD3 using α_vc_ = 0.1 and 0.02 shown in Figure 3a. It can be seen that the effective cavitation region increases significantly for α_vc_ = 0.02. The contours of the effective cavitation region 0.02 ≤ α_v_ < 0.6 for different designs are shown in Figure S1 (SI), and the variation of the effective cavitation region in terms of the iso-surface of α_vc_ = 0.02 at Δ*P* = 50, 150, 200, and 250 kPa for VD3 is shown in Figure S2 (SI). The predicted values of localized intense energy dissipation rates due to cavity collapse (ε_cav_) were obtained using the ANN-based surrogate model as a function of initial cavity radius for α_vc_ = 0.1 and 0.02 and are shown in Figure 3b. For α_vc_ = 0.02, the ε_cav_ values are similar for initial cavity radius values from 15 to 30 μm, whereas a larger difference in ε_cav_ values was observed for α_vc_ = 0.1. The reduced sensitivity to the value of the initial cavity radius is desirable since it is extremely difficult to accurately estimate the initial cavity radius. The influence of changing the effective cavitation region (α_vc_ = 0.02) on simulated DSD at varying pressure drops is shown in Figure 3c. For α_vc_ = 0.1, under the boundary condition of Δ*P* = 50 kPa, the second peak with a higher mean droplet diameter is predicted, but the first peak with a mean droplet diameter of ∼1 μm is nonexistent, whereas by using α_vc_ = 0.02, both the peaks are reasonably captured. For higher values of pressure drop, Δ*P* = 250 kPa, the mean droplet diameter corresponding to the first peak was identified correctly with the CFD+PBM simulation, although the second peak was not observed. The absence of a second peak in the DSD is due to the increased effective cavitation region with α_vc_ = 0.02. Considering these, the effective cavitation region defined by α_vc_= 0.02 was used in this work to examine the influence of device designs on flow and emulsification. These results are discussed in Section 2.2.2.

**Figure 3:**
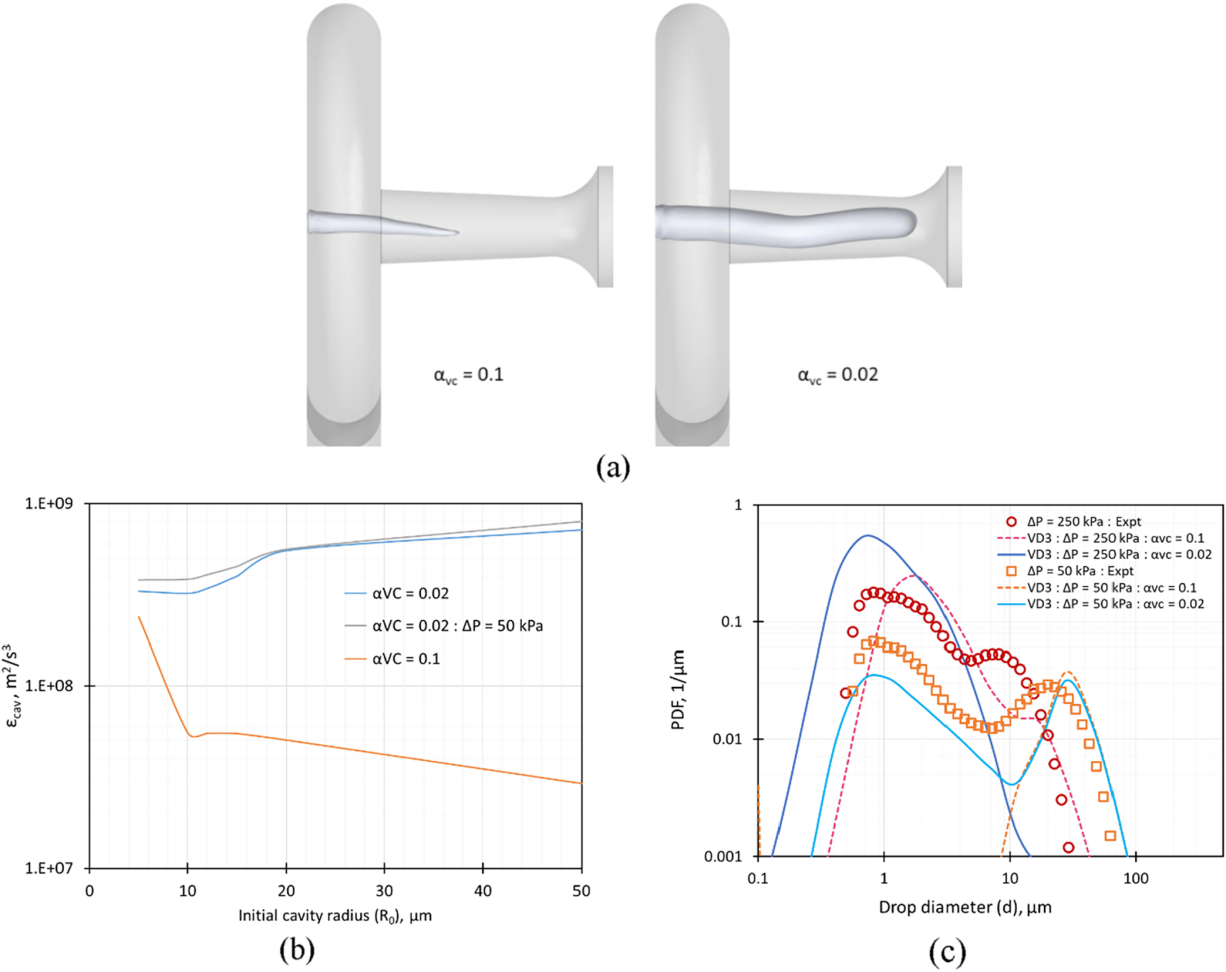
Influence of variation in α_vc_ on (a) effective cavitation region for the standard VD3 design at Δ*P* = 250 kPa, (b) ε_cav_ as a function of initial cavity radius, and (c) simulated DSD for VD3 design at Δ*P* = 250 and 50 kPa and comparison with the experimental value.

### 2.2 Shortlisting Devices for Experiments

#### 2.2.1 Comparison of Flow Features

Before simulating emulsification performance, the flow and cavitation characteristics of different VD design modifications were simulated and compared based on the strength of the vortex generated, the extent of cavitation, and the overall pressure loss in the devices. The strength of the swirl flow generated in the device impacts the minimum pressure value in the device, which defines the cavitation inception pressure and influences the extent of cavitation. The maximum tangential velocity, *V*_θmax_, or the ratio of *V*_θmax_ and velocity at the throat (*V*_T_), called as the swirl ratio (*V*_θmax_/*V*_T_), was used to characterize the strength of the vortex in the device. The value of maximum tangential velocity was obtained on a center line in the vortex chamber. The extent of cavitation, *C*_E_, is represented as the ratio of the amount of vapor generated (*V*_vap_) and the device volume (*V*_D_) as

1 $$C_{E}=\left(\frac{V_{\mathrm{vap}}}{V_{D}}\right)$$

The vapor volume is obtained by integrating the vapor fraction in a cylindrical region of diameter equal to throat diameter *d*_T_ and length three times *d*_T_.^27^ The energy consumption is defined as the product of pressure drop and flow through the device and can be characterized using the Euler number as

2 $$\mathrm{Eu}=\frac{\Delta P}{\frac{1}{2}\rho V_{T}^{2}}$$

where Δ*P* is the pressure drop across the device. The simulated results of different designs were then compared using these characteristics. Before such comparison, the simulated pressure drop values from CFD were validated by comparing experimental data obtained for different devices.

The comparison of simulated and experimental pressure drop results obtained for varying throat velocity for different VD designs (VD3, VD6, VD3_4I, and VD3_4N) is shown in Figure 4. These four devices were selected since they exhibited maximum variation in pressure drop characteristics. Barring the exception of VD3_4I, the difference in simulated and experimental values of the average Euler number for the different designs was found to be ∼10%. The coefficient of determination (*R* ^2^) was found to be 0.98. The relative root-mean-square error (RRMSE) was 0.18, indicating a slight shift in the simulation predicted pressure drop values as compared to the experimental data. The high *R* ^2^ (0.98) and low RRMSE values confirm that the CFD model accurately captures the pressure flow characteristics across the different VD designs and indicates the validity of the computational approach and the model assumptions considered here.

**Figure 4:**
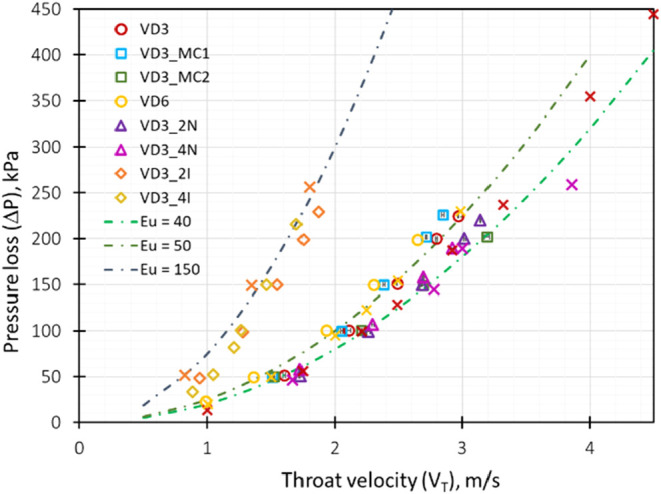
Comparison of experimental pressure drop versus throat velocity data with simulation results for different devices. The cross symbols represent simulation results; dotted lines represent the values corresponding to the Euler number, and experimental data are shown using open symbols.

Following the earlier work,^27^ the simulated Euler number and generated vapor were examined in terms of the swirl ratio and *V*_θmax_. The influence of design modifications on the simulated values of the Euler number and extent of cavitation is shown in Figure 5a,b, respectively.

**Figure 5:**
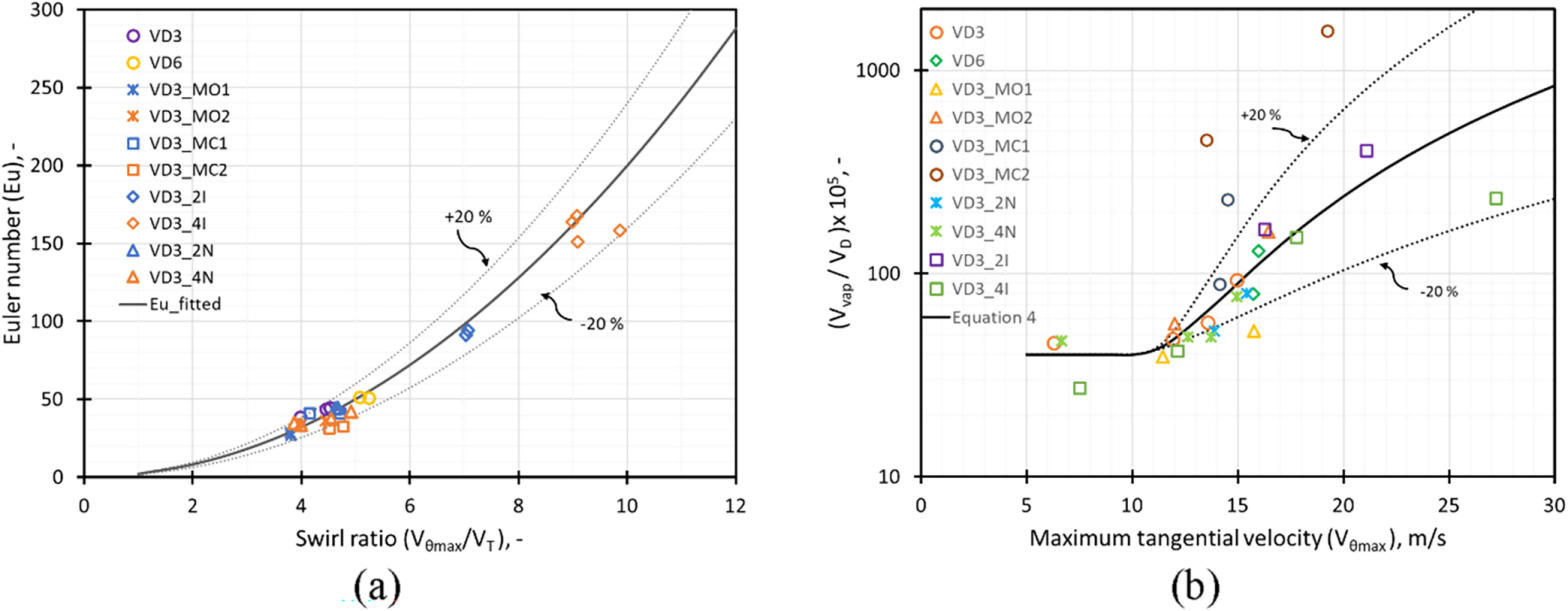
(a) Variation of simulated pressure drop values (Euler number, *Eu*) with the swirl ratio (*V*_θmax_/*V*_T_) and (b) variation of nondimensional vapor volume (*V*_vap_/*V*_D_) with maximum tangential velocity (*V*_θmax_) for different devices.

It is evident from Figure 5a that Eu follows a parabolic relationship with the swirl ratio as

3 $$\mathrm{Eu}={2\left(V_{\theta \max}/V_{T}\right)}^{2}$$

All the design modifications in this study follow the relationship given by eq 3 (with ±20% deviations shown by dotted lines in Figure 5a). For constant pressure drop conditions, the Euler number for multiple inlet devices showed an increase with the number of inlets as shown in Figure S3a (Supporting Information). This could be attributed to the increase in the flow rate and inlet area for these devices to achieve the same pressure drop value. The other devices with a single inlet showed a variation in the Euler number in the range of 30–50 with the least Euler number value observed for VD3_MO1. The variation in the swirl ratio (*V*_θmax_ */V*_T_) followed a pattern similar to that of the Euler number with an average value of 4.5 for devices with a single inlet. The multiple inlet devices showed an increase in the swirl ratio with the number of inlets with the maximum value ≈10 for a four-inlet VD (VD3_4I). The constant inlet throat velocity condition showed a trend similar to that of the constant pressure condition (Figure S3b of the Supporting Information).

The variation of nondimensional vapor volume with maximum tangential velocity for different devices shows a similar trend with the devices VD3_MC2 and VD3_MC1 showing relatively higher values. This is indicative of an early inception in these devices compared with the other devices.

It can be seen from Figure 5b that the critical limit of *V*_θmax_, beyond which vapor generation increases significantly, was found to be ∼10 m/s for all the considered devices. The following relationship of the extent of cavitation with the maximum tangential velocity was observed

4 $$C_{E}=\frac{V_{\mathrm{vap}}}{V_{D}}=a{\left(V_{\theta \max}-V_{\theta c}\right)}^{b}+c$$

where *V*_θ*c*_ is the critical tangential velocity at which cavitation inception occurs. The *V*_θ*c*_ was found to be ∼10 m/s, and the values of the constants *a*, *b*, and *c* were found to be 2, 2, and 40, respectively (with ±20% deviations in the constants *a* and *b* to accommodate most of the design modifications). The design with a change in the chamber shape was the outlier, with higher values of cavitation extent observed for this design, which could be the result of early inception. The device VD3_MO1 shows low values of *C*_E_ due to the expanded outlet throat section, which prohibits the complete development of the swirling flow. The increase in capacity or throughput with scale-up, scale-out, or multiple inlet configurations shows minimal impact on the values of *C*_E_. For the VD3_4I design, the increase in *C*_E_ with *V*_θmax_ was observed to plateau at higher values of *V*_θmax_. The vapor volume per unit device volume (*C*_E_) for different devices is shown in Figure S4 (Supporting Information) for the same pressure drop and the same inlet throat velocity conditions. The change in the chamber design to a pump casing/volute design was shown to have the maximum vapor volume for constant pressure drop conditions. The results for VD3_MC2 appear to disagree with the scaling relationship of *C*_E_ varying as (*V*_θmax_ – *V*_θ*c*_)^2^ as it performed quite differently from the other devices considered in this work. It was observed that it generated a significantly larger cavitation zone with vapor pockets present at the base of the chamber, which was not observed in other designs. Thus, the device VD3_MC2 was excluded from the development of the scaling law. While the relationship *(V*_θmax_ – *V*_θ*c*_)^2^ accurately captures trends for designs with similar chamber topologies (within ±20%), significant geometric modifications are not accounted for in the scaling law. The proposed scaling law is most robust when comparing reactors with the same fundamental chamber shape.

After comparing these lumped flow and cavitation characteristics for different devices, detailed flow fields (profiles of tangential velocity and development of swirl, cavitation zone, etc.) were examined for different design modifications. These results are discussed by organizing into four subparts dealing with modifications in the outlet port, vortex chamber shape, scale-up/scale-out, and the number of inlets.

##### 2.2.1.1 Influence of the Outlet Port Design

The influence of outlet throat-related modifications on the swirl ratio on a center line in the chamber, turbulent dissipation rate, and vapor fraction on a midplane across the chamber is shown in Figure 6a–c, respectively. The design modification represented by VD3_MO1 where the expansion ratio of the outlet throat diameter is increased 2.5 times shows a decrease in the swirl ratio. A decrease in the swirl ratio leads to a decreased magnitude of angular momentum in the vortex chamber. This implies that the pressure is not decreasing significantly in the core region and the cavitation zone is reduced. On the other hand, for the VD3_MO2 design, the swirl ratio values are similar to the standard VD3, indicating a similar performance in terms of cavitation extent, although the region of vapor present in the device (VD3_MO2) was seen to be reduced as the outlet nozzle was closer to the outlet throat as compared to the VD3 (see Figure 6c). The regions of high turbulent dissipation rates, where droplet breakage would be dominated by turbulent shear, were observed to be qualitatively similar to VD3 with the difference being that for VD3_MO1 and VD3_MO2, this region was present close to the outlet throat. This leads to a decreased breakage probability of oil droplets as this high-turbulent dissipation region was reduced due to modifications in the outlet region.

**Figure 6:**
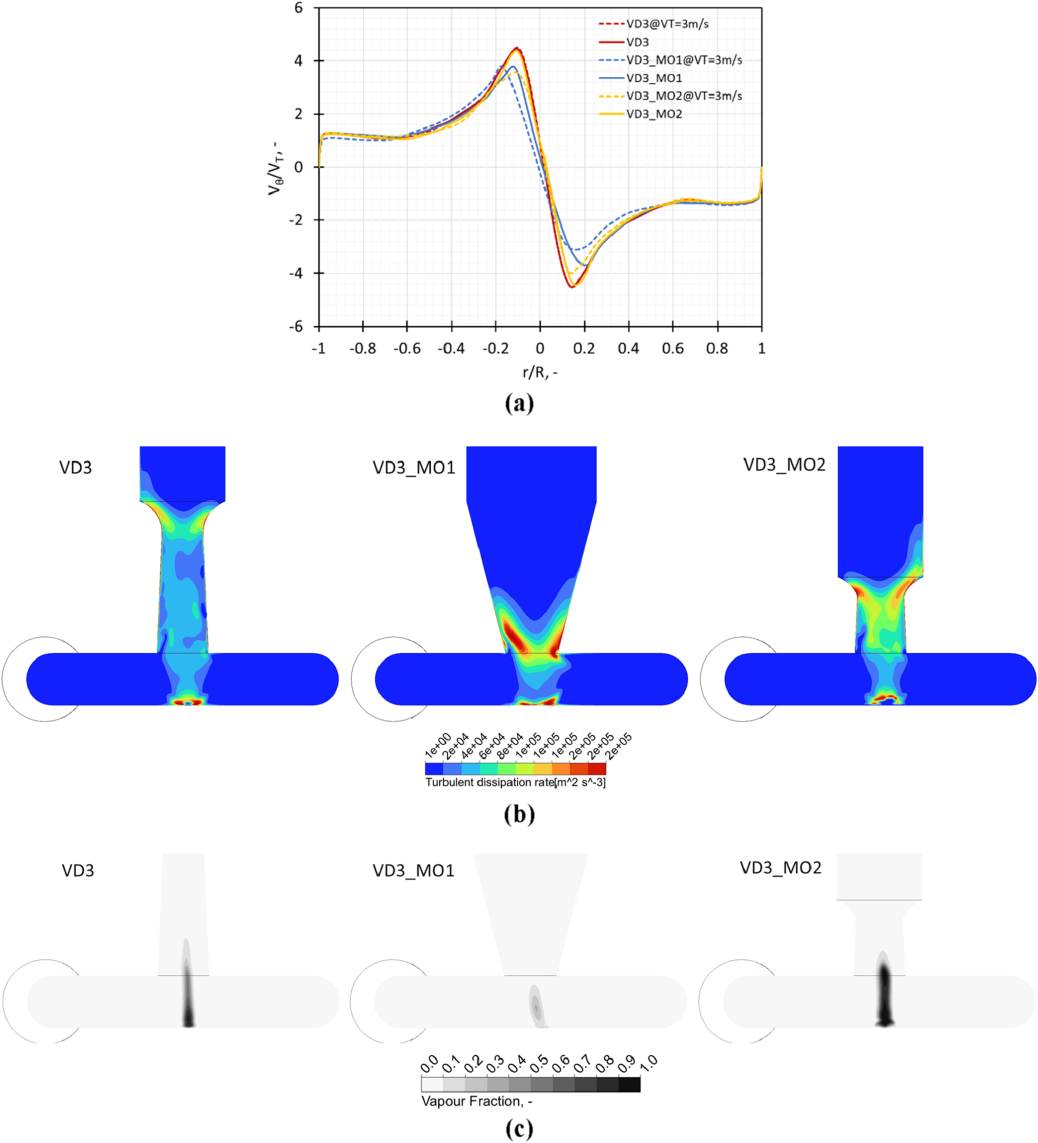
Influence of modifications in the outlet throat dimensions on (a) swirl ratio (*V*_θ_/*V*_T_) obtained on a midline in the vortex chamber at *V*_T_ = 3 m/s and Δ*P* = 250 kPa, (b) turbulence dissipation rate, and (c) vapor volume fraction on a midplane across the chamber.

##### 2.2.1.2 Influence of the Chamber Shape

The simulated results for devices with different chamber shapes are listed in Figure 7. The presence of a vortex stabilizer in the chamber was found to lead to a symmetrical distribution of the swirl ratio across the midline in the chamber (see Figure 7a). The swirl ratio (*V*_θ_/*V*_T_) values for the design modifications were on par with those observed for the standard VD3 design. This implies that the pressure reduction in the core region leading to the formation of cavitation zone was present in VD3_MC1 and VD3_MC2 devices like the VD3 design. The location of the point of maximum tangential velocity was similar for the VD3 and VD3_MC1 designs, while this point was seen to move further away from the center for the VD3_MC2 design. The turbulent dissipation rate contours for VD3_MC1 and VD3_MC2 were compared with those for VD3 and are shown in Figure 7b. The inclusion of a vortex stabilizer led to higher values of turbulent dissipation rate compared to VD3, whereas the change in the chamber shape to a pump volute led to lower values and a reduced region of turbulent dissipation rate. The higher values of turbulent dissipation rate observed for VD3_MC1 indicate increased breakage due to turbulent shear compared to VD3. The vapor volume fraction contours show that qualitatively, the region of a higher value of vapor volume fraction was larger in VD3_MC1 as compared to the standard design VD3. A similar observation was observed for the design with a volute or pump casing as the chamber (VD3_MC2) as shown in Figure 7c. The region with high values of turbulent dissipation rate is observed in the VD3_MC1 (VD3 with a vortex stabilizer) design, which could provide a higher breakage due to turbulent shear in this region. The breakage due to cavitation-induced turbulent shear could be higher in the VD3_MC2 design due to the increased cavitation extent for this device, although the breakage would also depend on the probability of the oil droplets encountering this region of high turbulent shear rate.

**Figure 7:**
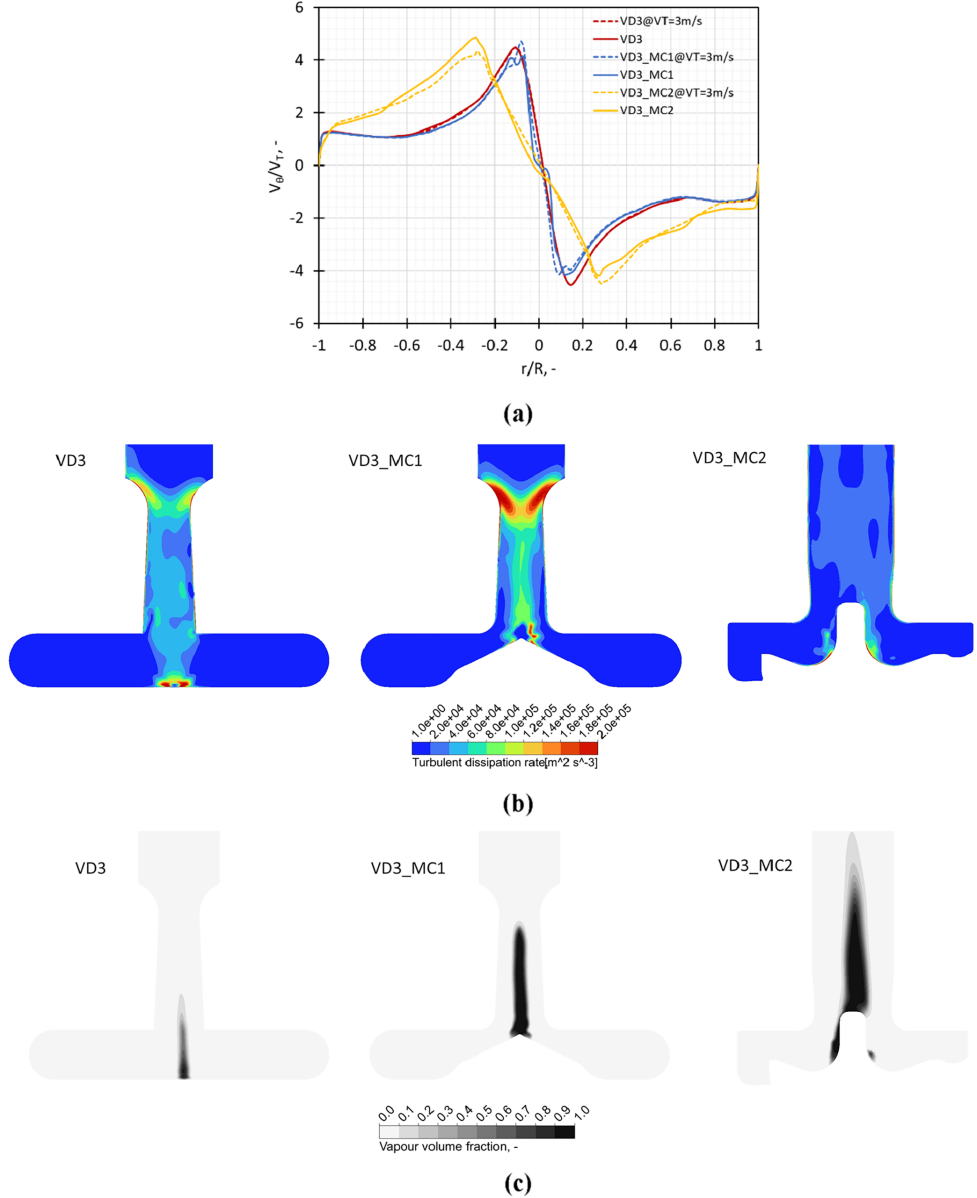
Influence of modifications in the outlet throat dimensions on (a) swirl ratio (*V*_θ_/*V*_T_) obtained on a midline in the vortex chamber at *V*_T_ = 3 m/s and Δ*P* = 250 kPa, (b) turbulence dissipation rate, and (c) vapor volume fraction on a midplane across the chamber.

##### 2.2.1.3 Influence of Scale-Up

The influence of scale-up on the swirl ratio on a center line in the chamber and vapor fraction on a midplane across the chamber is shown in Figure 8a and c, respectively. The geometric scale-up of the standard VD3 design leads to the VD6 design. The variation in the swirl ratio (*V*_θ_/*V*_T_) values at constant pressure drop and constant throat velocity conditions at a midline in the chamber showed no significant difference with scale-up of the standard design. While the Reynolds number increases with scale at constant velocity, the mean turbulent dissipation rate (ε̅) reduces with scale (or throat diameter of the device, *d*_T_) as

5 $$\mathrm{\varepsilon ̅}=\frac{\Delta PQ_{D}}{\rho V_{D}}$$

where Δ*P* is pressure drop across VD, *Q*_D_ and *V*_D_ are the flow rate through and volume of the VD, and ρ is the density of the liquid. For the same throat velocity, the pressure drops across VD3 and VD6 are almost the same (within 15%). The value ε̅ for VD6 is therefore lower compared to VD3. This is reflected in attenuated turbulence dissipation with VD6 compared to VD3 as seen in Figure 8b.

**Figure 8:**
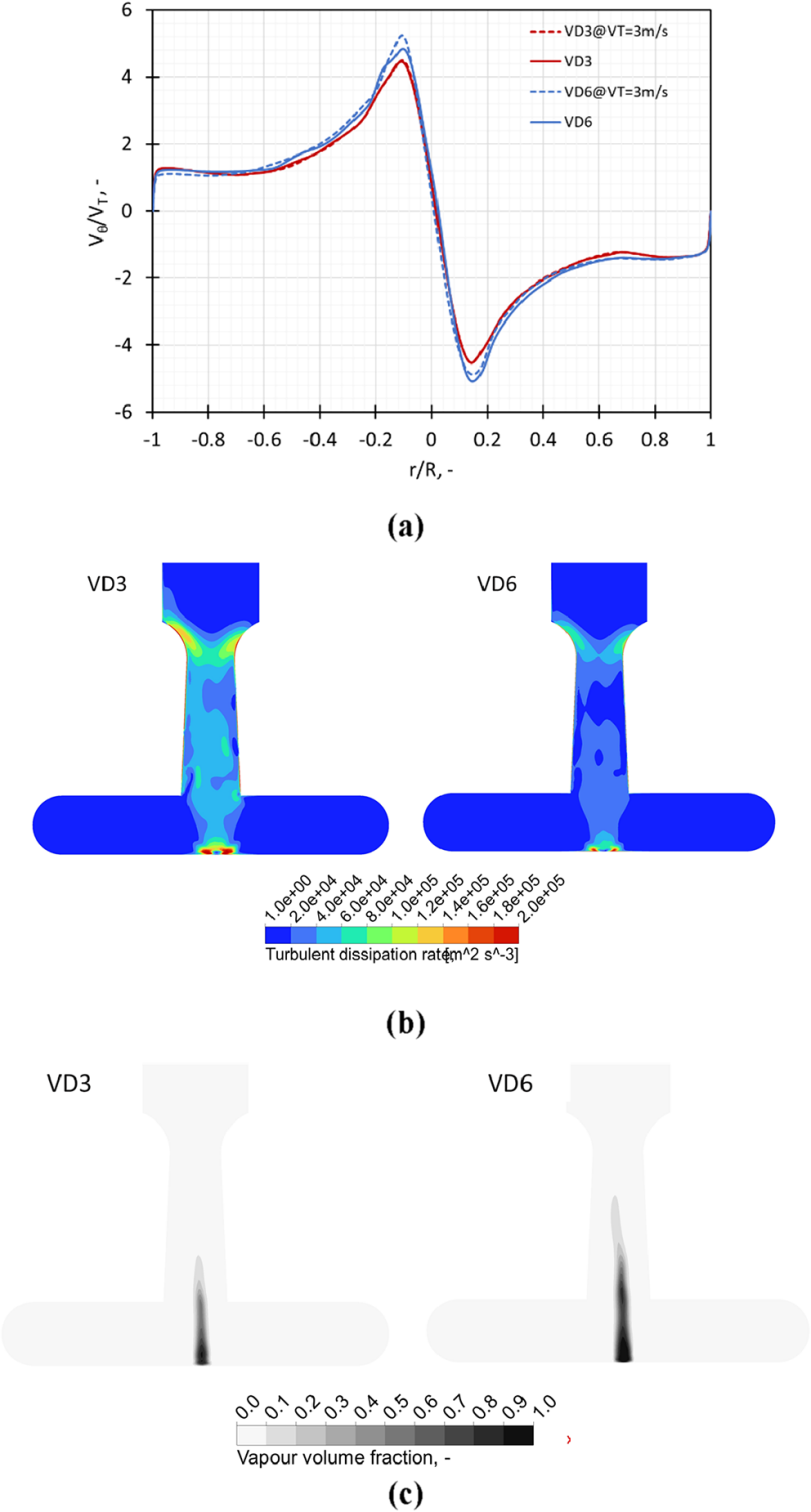
Influence of modifications in the outlet throat dimensions on (a) swirl ratio (*V*_θ_/*V*_T_) obtained on a midline in the vortex chamber at *V*_T_ = 3 m/s and Δ*P* = 250 kPa, (b) turbulence dissipation rate, and (c) vapor volume fraction on a midplane across the chamber.

The contours of turbulent dissipation rate on an axial midplane in the vortex chamber indicate that for VD3, a region with higher values of turbulent dissipation rate was more pronounced as compared to the scaled-up design VD6, but the region of cavitation or cavitation extent was higher for VD6 (Figure 8b). The increased region of higher turbulent dissipation rate in VD3 indicates a higher droplet breakage activity due to turbulent shear as compared to VD6, and a larger cavitation region in VD6 indicates increased probability of droplet breakage due to cavitation. These are contradictory observations, and the overall breakage due to the combined effect of turbulent shear and cavitation-induced turbulent dissipation rate could lead to similar breakage in these two devices.

##### 2.2.1.4 Influence of Scale-Out

The scale-out was accomplished by connecting the devices in parallel, as shown in Figure 1g,h. The influence of scale-out on the swirl ratio on a center line in the chamber and turbulent dissipation rate and vapor fraction on an axial midplane across the chamber is shown in Figure 9a–c, respectively. On increasing the number of devices in parallel, the performance in terms of the swirl ratio was observed to marginally decrease due to the increased connections present for operating the devices in parallel. The overall performance with respect to the distribution of turbulent shear rate and the cavitation region was observed to remain the same with an increase in the number of devices.

**Figure 9:**
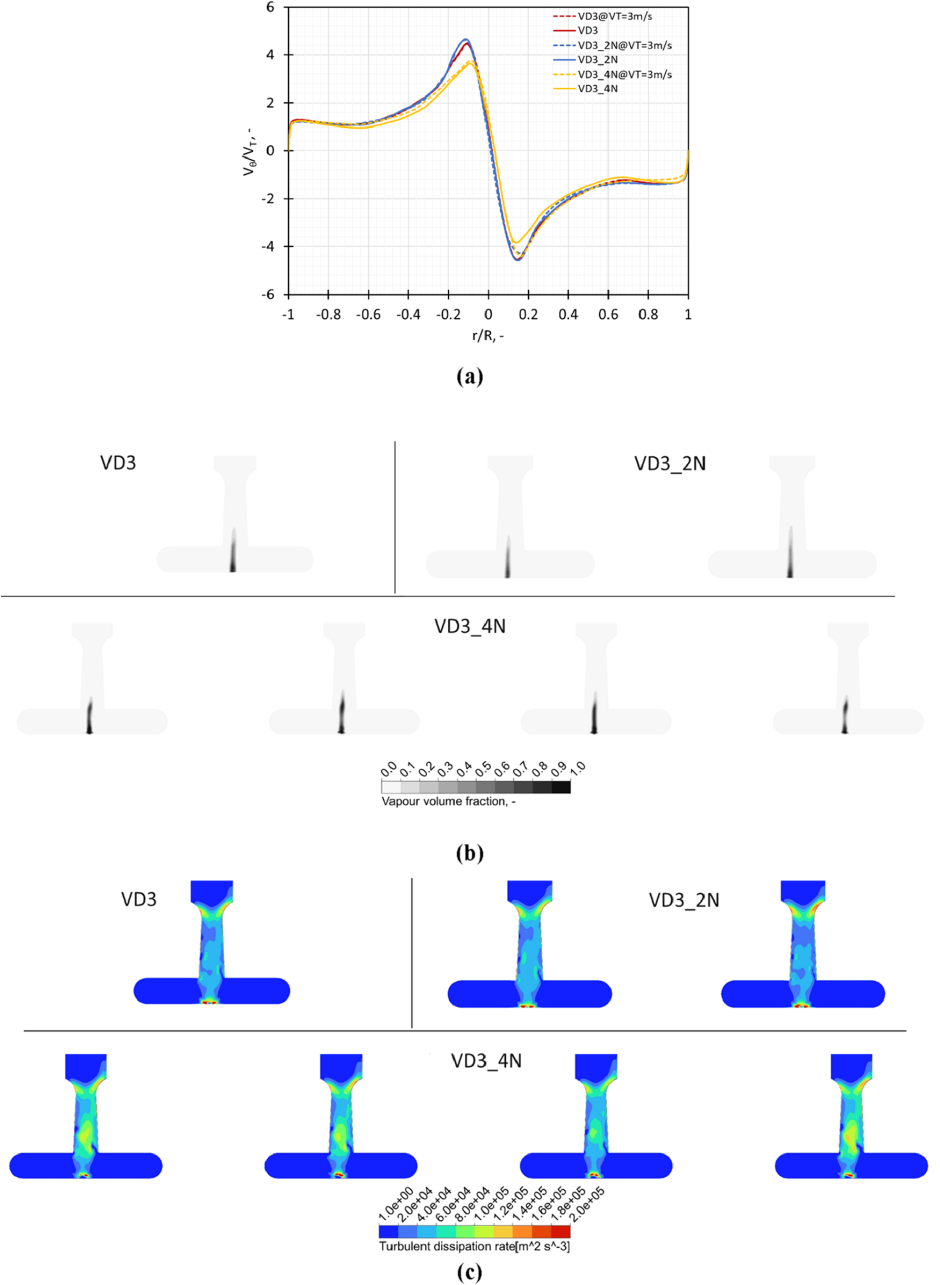
Influence of modifications in the outlet throat dimensions on (a) swirl ratio (*V*_θ_/*V*_T_) obtained on a midline in the vortex chamber at *V*_T_ = 3 m/s and Δ*P* = 250 kPa, (b) turbulence dissipation rate, and (c) vapor volume fraction on a midplane across the chamber.

##### 2.2.1.5 Influence of Multiple Inlets

The investigation of the multiple inlet designs was directed toward providing an additional option for increasing the capacity/throughput of the emulsion by maintaining a similar design as the standard one and to check if increasing the number of inlets leads to an improved performance at similar or lower power consumption values compared to the standard VD3 design. The increase in the number of inlets leads to increased swirl ratio values with the maximum value reaching around 10 for the VD3_4I design (Figure 10a). For constant pressure conditions, the increase in turbulent dissipation rate with the number of inlets is shown in Figure 10b and could be attributed to the increased flow rate in the devices. The increased flow rate at constant pressure condition also leads to an increase in the cavitation region or formation of more vapor due to cavitation for the multiple inlet devices. The translation of this increased performance to droplet breakage is investigated experimentally and discussed in Section 5.

**Figure 10:**
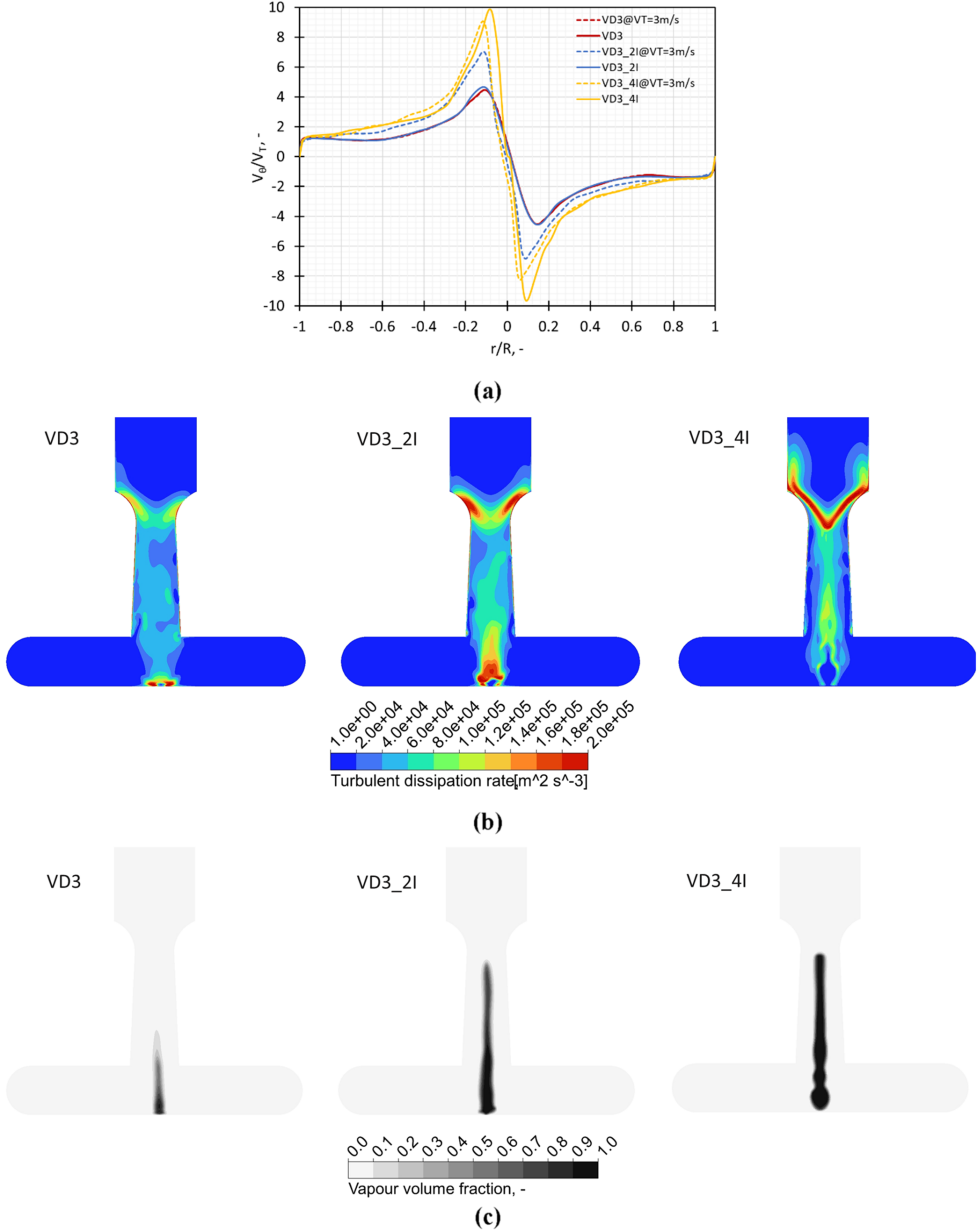
Influence of modifications in the outlet throat dimensions on (a) swirl ratio (*V*_θ_/*V*_T_) obtained on a midline in the vortex chamber at *V*_T_ = 3 m/s and Δ*P* = 250 kPa, (b) turbulence dissipation rate, and (c) vapor volume fraction on a midplane across the chamber.

#### 2.2.2 Comparison of Sauter Mean Diameters (*d*_32_)

The flow and cavitation performance of the different devices influences the droplet breakage in the VD. CFD+PBM simulations at a pressure drop value of 250 kPa were performed to simulate the DSD and Sauter mean diameters for the different designs. The simulated emulsification performance in terms of normalized Sauter mean diameter of the different design configurations is shown in Figure 11a,b. The normalization was presented with respect to the Sauter mean diameter of the standard VD3 design. Other relevant simulated results for different devices at Δ*P* = 250 kPa and flow rate ratio (*Q*_D_/*q*_net_) = 10 (flow rate, Euler number, swirl ratio, vapor generated, energy dissipation rates due to collapsing cavities and the Sauter mean diameter) are summarized in Table 2.

**Figure 11:**
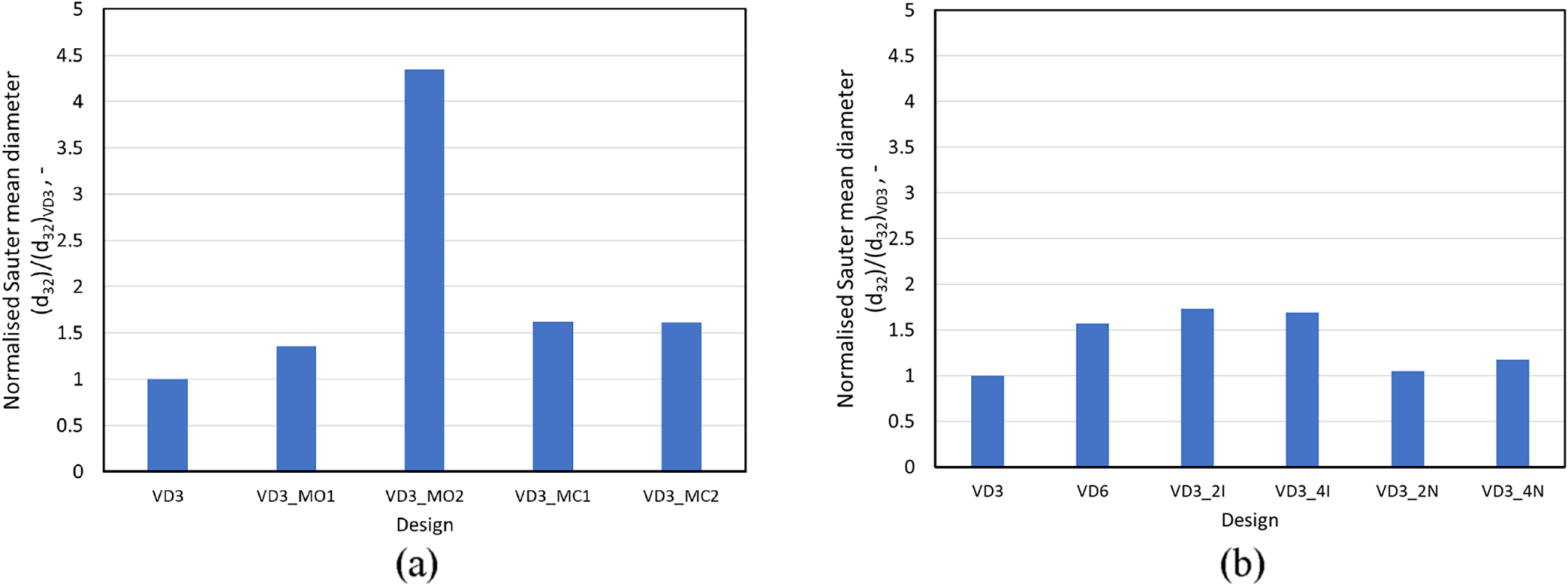
Comparison of normalized Sauter mean diameter (*d*_32_/(*d*_32_)_*VD*3_) for device modifications showing (a) influence of outlet configuration and chamber shape and (b) influence of capacity: scale-up, scale-out, and multiple inlet configurations.

**Table 2:** Summary of Simulation Results for Devices Operated at Δ*P* = 250 kPa and *Q*_D_/*q*_net_ = 10

device names	nominal flow, LPM	Euler number (*Eu*), -	max. swirl ratio *V*_θmax_/*V*_T_, -	volume of cavitation zone, m^3^ × 10^–8^	volume of vapor, m^3^ × 10^–10^	ε_cav_, m^2^/s^3^ × 10^8^	*d*_32_, μm
VD3	1.40	44.8	4.5	1.4	8.3	4.5	1.21
VD3_MO1	1.76	28.7	3.8	1.1	5.3	2.4	1.63
VD3_MO2	1.57	36.3	4.4	1.2	8.9	0.57	5.24
VD3_MC1	1.48	41.4	4.1	1.4	12	3.2	1.95
VD3_MC2	3.50	32.8	4.9	14	77	4.7	1.94
VD6	5.34	51.3	5.1	13	95	5.6	1.89
VD3_2N	2.80	44.7	4.7	2.6	14	4.8	1.26
VD3_4N	6.55	34.7	3.9	5.3	26	4.8	1.42
VD3_2I	1.95	94.2	7.1	1.8	14	4.8	2.09
VD3_4I	3.05	158.4	9.9	2.0	13	1.8	2.04

It can be seen from Figure 11a that the VD3_MO1 design showed an increase in *d*_32_, which seems to complement the flow performance observed for this device and discussed in Section 2.2.1 and quantitatively shown in Table 2. The swirl ratio (see Table 2) and volume of vapor in the effective cavitation region per unit volume of effective cavitation region was observed to decrease by around 15% for VD3_MO1 as compared to VD3. The VD3_MO2 design showed an increase of ∼4.5 times in normalized *d*_32_. This increase is largely due to the reduced length of the outlet port decreasing the probability of droplets to encounter the collapsing cavities in the effective cavitation region. The flow performance in terms of maximum swirl ratio and nondimensional volume of vapor in the effective cavitation zone shows the flow performance of VD3_MO2 at par with VD3, indicating that the droplet breakage in the effective cavitation region is dependent on the probability of the oil droplets passing through this region. Based on these considerations, the modifications to the outlet throat do not look promising for improving emulsification performance and therefore were not considered for further experimental evaluation.

The influence of the chamber shape on *d*_32_ shows that the Sauter mean diameter increases by ∼1.5 times for both cases (VD3_MC1 and VD3_MC2). The flow performance data (Table 2) show similar values of the swirl ratio for VD3_MC1 and VD3_MC2 compared to VD3, although an increase in the volume of vapor generated was observed for VD3_MC2 (compared to VD3). It should be noted that droplet breakage performance is determined by the intensity of cavity collapse (or, in other words, the energy dissipation rate induced by cavity collapse in the cavitation zone, ε_cav_) and probability of droplets encountering cavity collapse. Although the volute-shaped modification in VD3_MC2 creates a larger “effective cavitation region” and larger ε_cav_ than the standard design (VD3), the actual breakage efficiency is lower because in the VD3_MC2 design, the particles tend to bypass the effective cavitation zone. The simulated droplet fractions (for droplets with mean droplet size, *d* = 28 μm) for the VD3 and VD3_MC2 are shown in Figure S5 (Supporting Information). It can be seen from this figure that unlike the standard design VD3, where the droplets are concentrated in the effective cavitation zone, in VD3_MC2, even if the cavitation zone is larger, the droplets bypass the cavitation region and are less likely to encounter ε_cav_. This is also indirectly evident in the simulated DSD (see Figure S5 of the Supporting Information) for VD3_MC2, which shows a much larger peak of droplets formed because of prevailing turbulent shear than ε_cav_.

The design configurations for increased capacity using scale-up and multiple inlets showed normalized *d*_32_ values ∼1.6 times those observed for VD3. An increase in the number of devices by using a scale-out strategy showed no significant difference in the *d*_32_ values as compared to the standard device VD3. For the geometric scale-up of VD3 to VD6, the DSD and the position of the first peak are similar (see Figure S7 of the Supporting Information). The *d*_32_ value of VD6 was observed to be larger due to the higher spread in DSD as compared to VD3. The multi-inlet devices show a higher value of Sauter mean diameter compared to VD3, although the flow performance and cavitation activity results indicate an improved performance. These devices need to be investigated experimentally to draw definitive conclusions on the influence of scale-up, scale-out, or multiple inlet configuration on the characteristics of emulsions produced using these devices. Based on the simulated flow performance and emulsion characteristics of the different devices, the following device modifications were considered for further experimental evaluation besides the standard VD3: modified chamber shape (VD3_MC1 and VD3_MC2), scale-up and scale-out (VD6, VD3_2N, and VD3_4N), and number of inlets (VD_2I and VD_4I). The experimental methodology and experimental results obtained for these different devices are discussed in the following.

## 3 Experimental Section

### 3.1 Setup and Process Details

The emulsification experiments were conducted in the continuous mode with an internal loop using different modifications of vortex-based HC devices. The experimental setup was similar to the one used by Gode and Ranade.^28^ The modification in the setup was with regard to the use of a recirculation pump depending on the flow rate and corresponding pressure drop requirements. A Seaflo 22 Series DC diaphragm pump for lower flow rates and Vevor FL-40 series DC diaphragm pump for higher flow rates were used to circulate the mixture of the circulating emulsion and the newly incoming feed streams in the loop arrangement (see Figure S8 of the Supporting Information). The total circulation loop volume dictates the residence time and the relationship between the flow rate ratio (*Q*_D_/*q*_net_) and the equivalent number of passes (used for batch processing). In the continuous emulsion experiments performed in this work, the total recirculation loop volume was inclusive of the device volume, the volume of the connecting tubing and fittings, and the volume of emulsion present in the gas disengagement section (refer Table S1, Supporting Information). This volume was different for the setup with a low nominal flow rate (circulation loop volume = 120 mL) as compared to the one with a high flow rate (circulation loop volume = 350 mL).

Oil-in-water emulsions were produced using rapeseed oil (sourced from Newgrange Gold, Tesco, Ireland) and DI water (from an Elga ultrapure water system). The DI water was premixed with the surfactant Tween 20 (MP Biomedicals, LLC, France) at a concentration of 2 wt % of the total mass of the mixture.^28^ The choice of a high surfactant-to-oil ratio was a deliberate experimental strategy to ensure operation within a coalescence-inhibited regime. By providing an excess of surfactant, droplet surfaces are saturated almost instantaneously upon breakage. This allows us to isolate the breakage kinetics driven by the VD geometry without the confounding effects of droplet–droplet coalescence. If the surfactant concentration was lower, the observed droplet size distribution (DSD) would be a net result of both breakage and coalescence, making it difficult to quantify the intrinsic efficiency of the device design itself. In our previous work,^18^ it was shown that with a surfactant concentration of 2%, the emulsion was stable for at least 74 days without any significant change in the DSD. This coalescence time scale is several orders of magnitude larger than the time scales of experiments conducted in this work. Based on these considerations and prior results, 2% concentration of Tween 20 was selected in the present work. This ensured the production of stable emulsions and provided data for validating the models and for comparing the relative performance of different devices. The presented results and relative performance of different devices are not dependent on the surfactant concentration, as long as it is adequate to avoid coalescence. Although typical surfactant concentrations used in industrial applications are lower than 2%, the surfactant concentrations used are adequate to prevent coalescence. The droplets spend a very short time in the cavitation zone (∼10^–2^ s^26^) and therefore have a very limited exposure to hydroxyl radicals. The Tween 20 surfactant also forms a hydrated interfacial layer, which acts as a barrier to radical transport^30^ to oil molecules. This maintains the chemical stability of oil-in-water emulsions generated by the VD. Mustard oil-in-water emulsions produced using ultrasonication were found to be chemically stable.^31^ The nutritional profile is preserved in cavitation-induced emulsions.^32,33^ Thus, emulsions produced using the VD under the operating conditions considered in this study can be considered chemically stable. A more detailed discussion of the physical and chemical stability of emulsions produced using the VD is included in Section S2.2 (Supporting Information).

The experiments were conducted at one oil volume fraction (α_o_ = 0.05) based on the previous work of Upadhyay et al.^16^ They investigated the performance of vortex-based HC devices for rapeseed oil-in-water emulsions with oil volume fractions (α_o_) of up to 0.6. It was found that the Sauter mean diameter is independent of the oil volume fraction up to 0.35. The difference in the Sauter mean diameter of the oil volume fraction of 0.3 and 0.05 is within 0.25 μm. Considering these results, we restricted the experiments used for validation and for evaluating relative performance to oil volume fraction of 0.05 to minimize materials required and waste generated through these experiments. The results and guidelines established in this work for emulsions with an oil volume fraction of 0.05 would be applicable to denser emulsions at least up to an oil volume fraction of 0.35. The temperature during emulsification was assumed to remain constant based on theoretical calculations. There are many studies reporting increase in temperature while conducting hydrodynamic cavitation experiments using the batch mode, including ours.^34,35^ However, in this work, we used the continuous mode. The potential temperature rise in this mode was found to be less than 3 °C even if the losses to surroundings are neglected. Considering this rather small potential temperature change, it was assumed to be negligible and not considered in the calculations.

The experiments were performed across a wide range of operating conditions to thoroughly evaluate the performance of each device geometry. The VD3, VD3_MC2, VD3_4I, and VD3_4N were operated at three pressure drop values of 50, 150, and 250 kPa, whereas the rest of the designs were operated at 50 and 250 kPa pressure drop values. This range was chosen to evaluate the designs, as the lower pressure drop values (50 kPa) were close to the onset of cavitation for the standard VD3 and the higher pressure drop value (250 kPa) was selected to account for optimum cavitation effects. The key characteristics for each device under study in terms of the Euler number (*Eu*), which is the ratio of pressure forces to inertial forces, are summarized in Table 2. The total system volume was varied based on the scale of the device(s) or the parallel arrangement used. Emulsion samples were collected from the outlet port after the system reached a verified steady state. The experimental data reported here were based on samples collected after at least five residence times.^28^ The DSD of the collected samples was analyzed using a Malvern MasterSizer 3000. The refractive index for rapeseed oil was set to 1.466. Water at room temperature (20 °C) was used as the dispersant medium. The errors related to the experimental setup were quantified by performing a few experiments three times, and these are included on measured DSD and Sauter mean diameter values wherever possible.

### 3.2 SLA-Based 3D Printing: Device Fabrication

The VD3 design was based on the design patent of Ranade et al.^36^ The design modifications considered in the present study are summarized in Table 1. The designs selected for experimental investigations were fabricated using SLA AM via a Formlabs Form 3 3D printer, to enable testing the complex, modified geometries. The SLA process was selected for its ability to create complex and intricate models with high feature resolution and surface finish, as well as high rigidity. The Formlabs Form 3 SLA 3D printer^37^ was used with a Formlabs Clear Resin V4 (product code: FLGPCL04), under high-resolution parameter settings, at a layer thickness of 0.05 mm and with a minimum feature size of 0.13 mm (with tolerances of ±0.05 mm). The images of devices fabricated via AM are shown in Figure S9 (Supporting Information). The devices were fabricated by using stereolithography (SLA)-based 3D printing. All printed components were postprocessed according to standard protocols, including solvent washing and UV curing, to ensure full polymerization and mechanical integrity. SLA allows for the fabrication of complex internal geometries that are difficult to fabricate otherwise. Postcured SLA resins have a high Young’s modulus (∼2.8 GPa), making them immune to deformation under the high liquid flow rates and pressures typically required for cavitation.

There is a possibility of potential microplastic generation when resin-based cavitation devices are used. The vortex devices investigated in this workgenerate a strong swirling flow and a low-pressure core. Therefore, cavitation predominantly occurs within the bulk fluid region rather than in the vicinity of solid surfaces. This significantly reduces the likelihood of cavitation-induced erosion. The primary objective of this work is to develop and validate a predictive modeling framework and to demonstrate its utility in optimizing device design. A detailed assessment of material compatibility and potential leaching or microplastic generation was therefore beyond the scope of this work. Nevertheless, given that (i) cavitation activity is localized away from solid boundaries and (ii) no visible erosion or surface degradation was observed during the experimental campaigns, the likelihood of significant microplastic generation is expected to be low under the conditions investigated. For industrial deployment, systematic studies on material durability, erosion resistance, and potential particle release will be valuable as part of future work. For applications requiring stringent purity standards, the optimized designs identified in this study can be readily implemented using industrial-grade materials such as stainless steel or titanium.

For devices fabricated using 3D printing technology, the roughness is an important property that affects the overall flow hydrodynamics. In the present study, the devices were fabricated using 3D printing with dimensional tolerances ±0.015 mm and show typical roughness (*R*_a_) values in the 0.3–2 μm range depending on layer height and build orientation.^38–40^ While the roughness height (*R*_a_) was not experimentally quantified, the influence of *R*_a_ on the Euler number for VD3 was evaluated using CFD simulation. The comparison of the simulated tangential velocity profile with smooth and *R*_a_ = 18 μm (an order of magnitude larger than actual SLA roughness) are quite small as shown in Figure S10 (Supporting Information). Since the actual roughness is of the order of 10° μm, the difference in *Eu* or swirl ratio will be insignificant. This was also confirmed by comparing the measured observed pressure drop values for the 3D-printed device with those of the CNC-machined device. The Euler number for both of these devices agreed within 1% (with the experimental error bars also observed to be within 1%).

## 4 Results and Discussion

The emulsification performance of devices shortlisted based on numerical investigations was experimentally investigated and compared in terms of emulsion characteristics (DSD and characteristic diameters: *d*_32_, *D*10, *D*50, *D*90) and emulsification energy efficiency (η). The energy efficiency (η) is defined as the ratio of minimum power required for new surface area generation to the actual power consumption and is given by the relation

6 $$\eta =\frac{6q_{\mathrm{net}}{\sigma \alpha }_{0}}{d_{32}P}100$$

where *q*_net_ is the net flow rate of emulsion, σ is the interfacial tension, α_0_ is the oil volume fraction, and *P* is the power consumption, given by

7 $$P=\Delta PQ_{D}$$

Here, Δ*P* is the pressure drop across the device and *Q*_D_ is the flow rate through the device. The emulsion characteristics of the different device design modifications and configurations were compared based on the energy consumption per kilogram of emulsion (*E*) produced and is given by

8 $$E=\frac{P}{\rho_{m}q_{\mathrm{net}}}$$

where ρ_m_ is the emulsion density. The energy consumption per kilogram of emulsion is seen to be proportional to the flow rate ratio $\left(\frac{Q_{D}}{q_{\mathrm{net}}}\right)$ .

### 4.1 Comparison of DSD and Sauter Mean Diameter (*d*_32_)

#### 4.1.1 Influence of the Chamber Shape

The influence of the chamber shape on the DSD at two pressure drop values of 50 and 250 kPa and flow rate ratio values of 10 is shown in Figure 12a. The DSD shows two peaks, which are attributed to two different mechanisms prevalent in the devices. The first peak with a lower mean droplet diameter (∼10° μm) corresponds to droplet breakage by cavity collapse, whereas the second peak appears due to droplet breakage by turbulent shear. The height of the first peak increases with an increase in pressure drop across the device, indicating increased breakage due to cavitation. The presence of the first peak at a pressure drop of 50 kPa points to the cavitation phenomenon being present for droplet breakage, however, significantly lower than at higher pressure drop. For the VD3_MC2 design, the height of the first peak is lower as compared to the standard VD3 and modified chamber VD3_MC1 design. For this design, the cavitation region was observed to be larger than those of the other designs (see Figure 7c). However, the lower height of the first peak indicates that the oil droplets bypass this cavitation-induced very high-turbulent dissipation rate region, leading to droplet breakage happening majorly due to turbulent shear. The DSDs at a lower pressure drop value are similar for the three designs with the VD3_MC2 showing comparatively lower first and second peaks as compared to the other designs.

**Figure 12:**
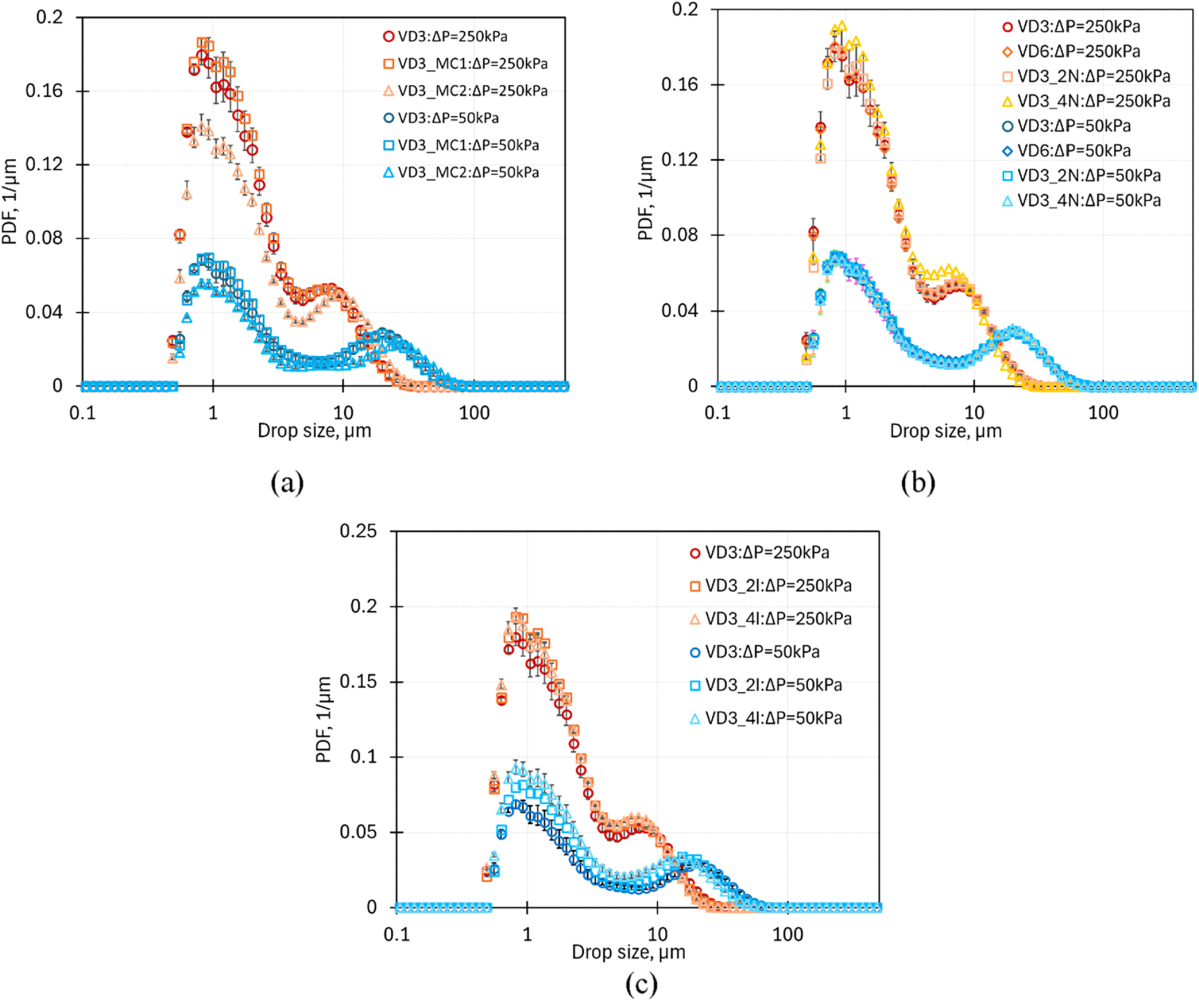
Comparison of DSD for devices with (a) change in chamber shape, (b) scale-up, scale-out, and (c) multiple inlet configuration.

The comparison of Sauter mean diameter, *d*_32_, for the designs with chamber shape modifications and the standard VD at Δ*P* = 50 and 250 kPa and varying flow rate ratios is shown in Figure 13a. The Sauter mean diameter was related to the energy consumption per unit mass of emulsion as

9 $$d_{32}=d_{321}E^{-0.2}$$

where *d*_321_ is the value of the Sauter mean diameter at *E* = 1 J/kg. The variation of *d*_32_ with *E* is shown in Figure 13a–c with the dashed lines representing eq 9, and the values of *d*_321_ are listed in Table 2 and are shown in Figure 13d. The values of *d*_321_ for VD3 and VD3_MC1 were equal, whereas for VD3_MC2, the value is higher, indicating a lower emulsification performance of VD3_MC2 compared to the standard VD3 or VD3_MC1 (see Figure 13d). The *d*_321_ values are seen to decrease with an increase in pressure drop as the cavitation activity increases, thereby allowing more droplets to encounter the collapsing cavities. At a higher pressure drop value (Δ*P* = 250 kPa), the change in *d*_32_ with *E* reduces as compared to a lower pressure drop (Δ*P* = 50 kPa). To achieve a desired *d*_32_, either the pressure drop could be increased or the net emulsion flow rate could be decreased at a lower pressure drop value. The variation of other characteristic diameters (*D*10, *D*50, and *D*90) and span with *E* is shown in Figure S12 (Supporting Information), and the quantitative values are tabulated in Table S2 (Supporting Information). The observed values of *D*10 for all the three devices were almost the same, whereas a variation is observed in the values of *D*50 and *D*90 for VD3_MC2, which implies that for this device, the larger droplets bypass the cavitating region and the drop breakage occurs by turbulent shear away from the cavitating region, leading to increased *D*90 and span. A similar value of *D*10 signifies that droplet breakage by cavitation generates a similar size of small droplets independent of the chamber shape. The reduction of *D*90, *D*50, and *D*10 with an increase in energy consumption is due to an increase in the shear and cavitation activity in the device along with increased probability of droplet breakage.

**Figure 13:**
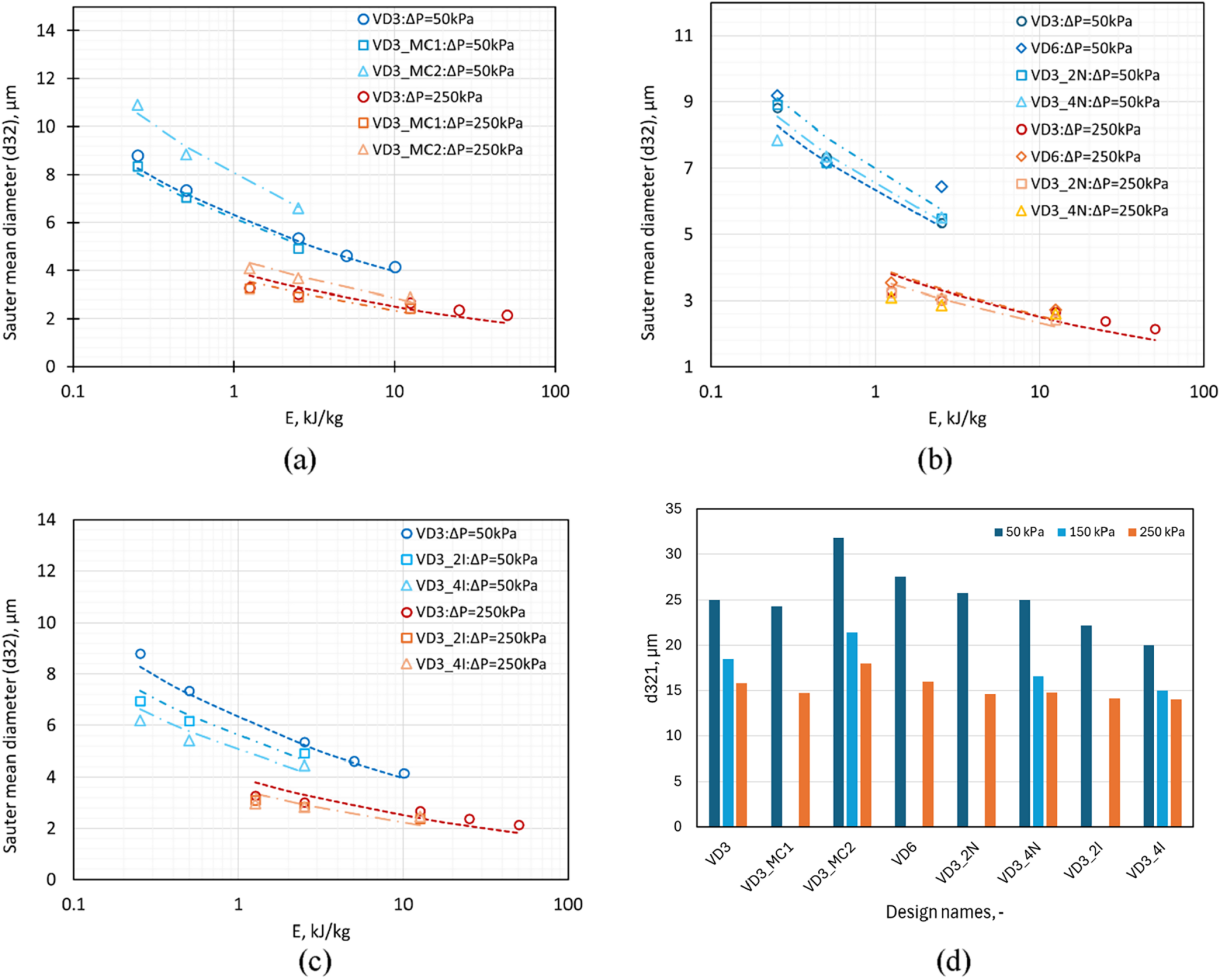
Comparison of *d*_32_ for devices with (a) change in chamber shape, (b) scale-up, scale-out, and (c) multiple inlet configuration. Dotted lines are based on eq 9 with *d*_321_ from Table 3. (d) Variation in *d*_321_ for different design modifications at varying pressure drop values.

#### 4.1.2 Influence of Capacity: Scale-Up, Scale-Out, and Multiple-Inlet Configurations

Similar to the observations with earlier results, the DSDs obtained for devices with scale-up, multiple inlets, and numbering up options show a bimodal nature. The mean droplet diameter corresponding to the first peak signifying breakage due to collapsing cavities was ∼10^0^ μm with no significant change observed for the different device modifications/configurations. The mean diameter corresponding to the second peak showed a small variation for the different designs depending on the turbulence dissipation rate in the device. The variation of DSD with scale-up and scale-out configurations is shown in Figure 12b for pressure drop values of 50 and 250 kPa and a flow rate ratio of 10. The DSDs are the same for the same value of pressure drop, indicating that the influence of geometric scale-up and numbering up was insignificant with regard to the emulsions produced. At higher pressure drop values, the contribution of droplet breakage due to collapsing cavities supersedes that due to turbulent shear so that the height of the first peak increases significantly. At a pressure drop value of 250 kPa, with an increase in *E*, the DSD moves toward monomodal (see Figure S13 of the Supporting Information), indicating the increase in droplet breakage due to cavitation and increased probability of droplets encountering collapsing cavities. The multi-inlet VDs (VD3_21 and VD3_4I) were seen to perform slightly better as compared to the VD3 design for continuous emulsification. For VD3_21 and VD3_4I, the DSDs at 250 kPa were similar with differences within the experimental error, whereas for a lower value of pressure drop (Δ*P* = 50 kPa), the heights of the first and second peaks were the highest for VD3_4I (see Figure 12c). The improved performance of VD3_4I (and VD3_2I) is predominantly due to the increased flow rate through the device for the same pressure drop value.

The Sauter mean diameter decreases with increasing *E* and is almost the same for the scale-up and scale-out configurations for a particular value of *E* as shown in Figure 13b. At lower pressure drop values, the value of Sauter mean diameter is slightly larger for VD6 as compared to VD3, which could be attributed to the turbulent energy dissipation rate distribution discussed above in Section 2.2.1, where a larger region with higher values of turbulent dissipation rate was observed for VD3 as compared to VD6. Figure S14 (SI) shows the influence of scale-up and scale-out of VD3 on other characteristic diameters (*D*10, *D*50, and *D*90) and span with *E*. In line with the observations for DSD and *d*_32_, it was observed that numbering up of VD3 shows insignificant differences in the *D*10, *D*50, and *D*90, whereas an increase in the scale of VD3 from 1 to 5 LPM (VD6) nominal flow rate led to a slight increase in *D*90. The decrease in *d*_32_ with an increase in the number of tangential inlets is observed at lower pressure drop values, although the difference becomes negligible at higher pressure drop values (see Figure 13c). This could also be inferred from the values of *d*_321_ in Figure 13d, which shows a higher difference at Δ*P* = 50 kPa. The values of *D*10 (see Figure S12 of the Supporting Information) are similar for the VD3 and multi-inlet devices, which further emphasizes the observation that droplet breakage due to cavitation produces similar smaller droplets and are independent of the design or configuration. Also, lower values of *D*50 and *D*90 for multi-inlet devices (VD3_2I and VD3_4I) point to increased turbulent shear because of the increased flow rate through the device for the same pressure drop value. Overall, the attainable *d*_32_ is a function of *E*, which may be achieved by different combinations of pressure drop (Δ*P*) and flow rate ratio (*Q*_D_ */q*_net_) (see eqs 7, 8, and 9) (Table 3).

**Table 3:** Values of *d*_321_ for Different Design Modifications at Varying Pressure Drop Values

Δ*P*, kPa → device names ↓	50	150	250
VD3	25	18.5	15.8
VD3_MC1	24.3		14.7
VD3_MC2	31.8	21.4	18.0
VD6	27.5		16.0
VD3_2N	25.8		14.6
VD3_4N	25	16.6	14.8
VD3_2I	22.2		14.1
VD3_4I	20	15.0	14.0

### 4.2 Comparison of Emulsification Energy Efficiency (η)

The influence of the chamber shape on the emulsification energy efficiency (η) for variation in *E* is shown in Figure 14a. The emulsification energy efficiency was observed to decrease with an increase in *E* and followed the relation given by

10 $$\eta =\eta_{1}E^{-0.8}$$

where η_1_ is the emulsification efficiency at *E* = 1 J/kg. Combining eqs 6 and 8–10, the relation between η_1_ and *d*_321_ could be arrived at as

11 $$\eta_{1}=\frac{6{\sigma \alpha }_{0}}{{\rho_{m}d}_{321}}100=\frac{1055}{d_{321}}$$

where *d*_321_ is in microns. The values of η_1_ for different devices at varying pressure drop values are shown in Figure 14d. At the same value of *E*, the energy efficiency of drop breakage (η) is higher at a pressure drop value of 250 kPa as compared to Δ*P* = 50 kPa, whereas this difference reduces for change in the pressure drop value from Δ*P* = 150 kPa to Δ*P* = 250 kPa. At Δ*P* = 50 kPa, the cavitation phenomenon is intermittent as the inception point is around 50 kPa, which leads to the lower breakage due to collapsing cavities, which significantly increases with the increase in the pressure drop value. The inclusion of a vortex stabilizer (VD3_MC1) led to a slight increase in energy efficiency as compared to the standard VD3 design, whereas for a design with the chamber shape as a pump volute (VD3_MC2), the energy efficiency decreased (>20% for Δ*P* = 50 kPa and >15% for Δ*P* = 250 kPa) as compared to the standard VD3 design as shown in Figure 14a.

**Figure 14:**
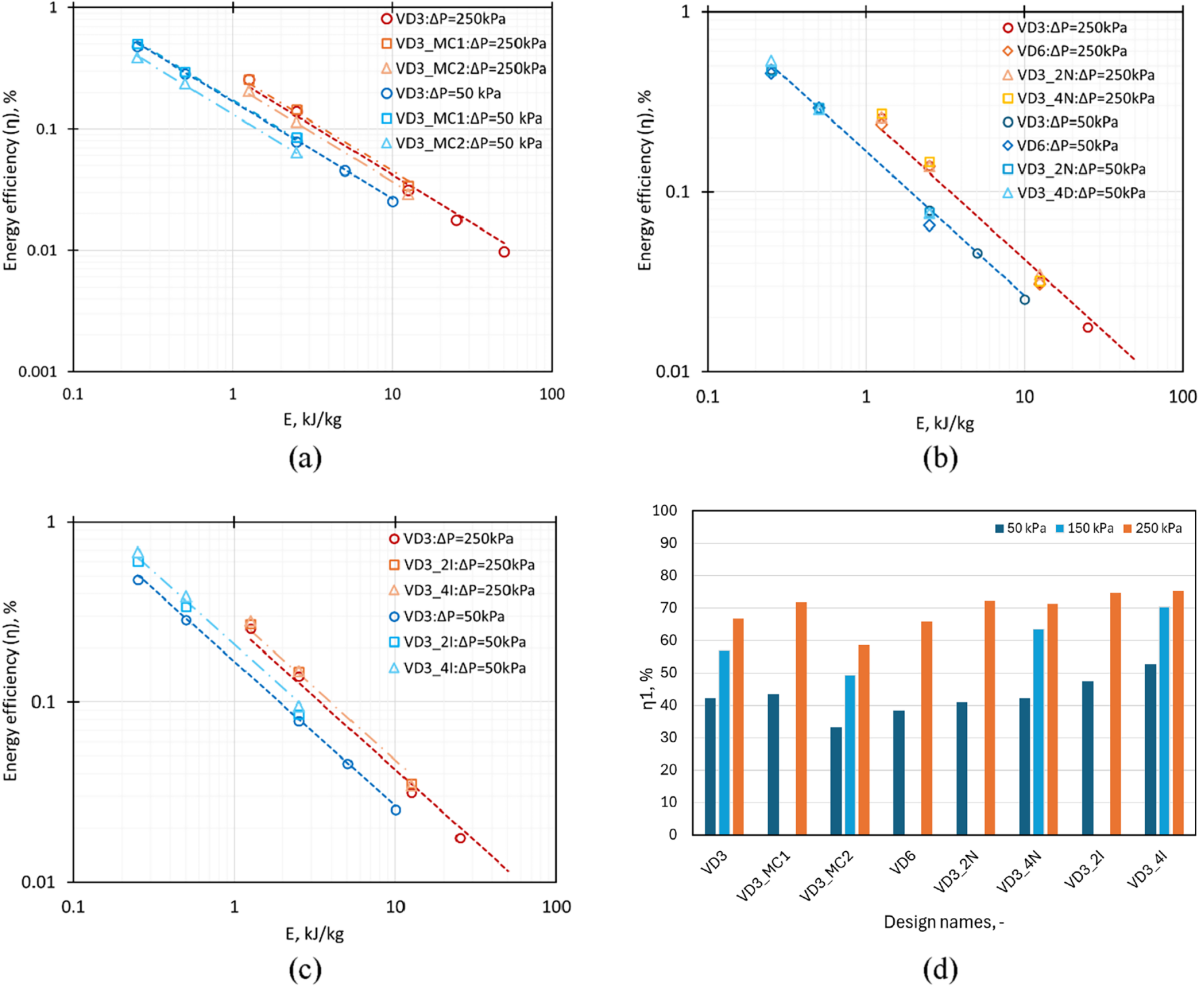
Comparison of η for devices with (a) chamber shape change, (b) scale-up and scale-out implementation, and (c) multiple tangential inlets and with eq 10 represented by a dotted line. (d) Variation in η_1_ (eq 11) for different design modifications at varying pressure drop values.

The variation of emulsification energy efficiency (η) with *E* at two different pressure drop values for scale-up and scale-out configurations and multiple tangential inlet designs is shown in Figure 14b,c, respectively. As observed for other design modifications, the η decreases with an increase in *E* and is represented by eq 10. The scale-up and/or scale-out configurations show similar values of energy efficiency as VD3. The multi-inlet devices (VD3_2I and VD3_4I) were observed to give a higher energy efficiency as compared to the standard VD3. The difference in energy efficiency values increases with an increase in the number of inlets (>25% for Δ*P* = 50 kPa and >15% for Δ*P* = 250 kPa: VD3_4I vs VD3) and decreased at higher pressure drop values. Additional continuous emulsification experiments were carried out at the same average turbulent energy dissipation rates (ε̅) for a standard device (VD3) and multi-inlet devices (VD3_2I and VD3_4I). The results (shown in Figure S16 of the Supporting Information) indicate that at the same value of ε̅, the devices show similar performance in terms of DSD, *d*_32_, and η.

Overall, the configuration with multiple inlets showed superior flow performance in terms of the swirl ratio and *C*_E_, which was complemented by improvements in the emulsification energy efficiency (more prominent at lower pressure drop values) at a reduced droplet size. At higher values of pressure drop (Δ*P* = 150–250 kPa), the difference between multi-inlet devices and the VD3 diminishes, in which case, the use of a scale-out configuration could be preferred to avoid the complicated connections needed for the multi-inlet devices. The scale-out configuration could be further designed to change the number of active devices based on the capacity requirements (see Figure S17 of the Supporting Information).

The presented results and approach would be useful to identify appropriate device and operating conditions (pressure drop or flow rate through the loop) based on the desired net emulsion flow rate, *q*_net_, and the desired Sauter mean diameter, *d*_32_. The CFD+ANN+PBM models (discussed in detail by Gode and Ranade^28^) and the guidance on the influence of device modifications presented here would be useful to develop newer designs of cavitation devices for a variety of applications.

## 5 Conclusions

The different design modifications of the vortex-based HC device were studied computationally and experimentally for the continuous production of rapeseed oil-in-water emulsions. The CFD+PBM+ANN simulation framework was used for comparing the flow and emulsification performance of different design modifications of vortex-based HC devices. The SLA-based additive manufacturing was used to fabricate the shortlisted devices for experimental investigations. The key conclusions based on the simulated and experimental results are

- For all the considered devices, Eu was found to scale with square of the swirl ratio (eq 3).
- The cavitation extent *C*_E_ was found to scale with (*V*_θmax_ – *V*_θ*c*_)^2^ for *V*_θmax_ > *V*_θ*c*_ ≈10m/s for designs with similar chamber topologies.
- The device with a pump casing/volute as the chamber shape exhibited the maximum value of *C*_E_. For all other considered devices, the value *C*_E_ was similar to the standard design VD3.
- The inclusion of a vortex stabilizer in the chamber shape led to improved cavitation performance as well as improved distribution of a high value of turbulent dissipation rate in the outlet throat region. The geometric scale-up and scale-out modifications showed insignificant differences in the cavitation and flow performance as compared to the standard device VD3.
- In the case of devices with multiple inlets, the swirl ratio increased with an increase in the number of inlets (at the same pressure drop across the device) and showed a maximum value of ∼10 for the device with four tangential inlets operated at 250 kPa pressure drop value.
- Irrespective of the device design or configuration, a bimodal DSD was observed at lower values of *E*, which moved toward a monomodal distribution for higher pressure drop values and increase in *E* or flow rate ratio.
- The Sauter mean diameter was found to be proportional to *E* ^–0.2^ (eq 9), and the droplet breakage efficiency was found to be proportional to *E* ^–0.8^ (eq 10).
- The change in the chamber shape to a pump volute led to a decrease in performance for continuous emulsification with higher values of *d*_32_ and lower values of η. The inclusion of a vortex stabilizer in the chamber shape showed slightly improved performance compared to the standard device.
- On using the scale-up or scale-out configurations, the emulsification performance remained the same as the standard device.
- With an increase in the number of tangential inlets, the device showed better performance compared to the standard device. This improvement, in terms of *d*_32_ and η, was prominent at lower pressure drop values and reduced at higher pressure drop values.

The presented approach, results, and guidelines for designing newer VD-based devices would be useful for developing optimized devices for a variety of applications besides emulsification.

## Supplementary Material



## Notes

CFD model equations, CFD+PBM+ANN simulation results showing comparison of effective cavitation region, DSD, photograph of a typical experimental setup and schematic of vortex-based hydrodynamic cavitation for continuous emulsification, images of SLA 3D-printed devices used in experiments, experimental data showing variation of characteristic diameters, and images of scale-out configurations to change the number of active devices (PDF)

## Nomenclature

*C*_E_: extent of cavitation
*D*10: droplet diameter at 10 vol % in the DSD curve, μm
*d*_32_: Sauter mean diameter, μm
*d*_321_: value of Sauter mean diameter at *E* = 1 J/kg, μm
*D*50: droplet diameter at 50 vol % in the DSD curve or median of DSD, μm
*D*90: droplet diameter at 90 vol % in the DSD curve, μm
*d*_T_: throat diameter, mm
*E*: energy consumption per unit mass of emulsions, J/kg
ER_o_: Expansion ratio of outlet port
*Eu*: Euler number
*k*: turbulent kinetic energy, m^2^/s^2^
*l*_o_: Length of outlet port nozzle, mm
*P*: power consumption, J/s
Δ*P*: pressure drop in the device, kPa
*Q*_C_: volumetric flow rate in the circulation loop, m^3^/s
*Q*_D_: volumetric flow rate through the emulsification device, m^3^/s
*q*_net_: net volumetric flow rate, m^3^/s
*R*_0_: initial cavity radius, μm
*V*: total system volume, ml
*V*_D_: volume of the vortex-based device, m^3^
*V*_vap_: volume of vapor generated in the device, m^3^
*V*_T_: throat velocity, m/s
*V*_θ_: tangential velocity on a midline in the chamber, m/s
*V*_θmax_: maximum tangential velocity on a midline in the chamber, m/s
*V*_θc_: critical value of maximum tangential velocity for cavitation inception, m/s
ω: specific dissipation rate, 1/s

## Greek Letters

α: vapor volume fraction
ε: turbulent energy dissipation rate, m^2^/s^3^
ε̅: average energy dissipation rate per unit mass in the device, m^2^/s^3^
η: energy efficiency for droplet breakage, %
η_1_: energy efficiency at *E* = 1 J/kg, %
μ: viscosity, kg/m s
ρ: density, kg/m^3^
σ: interfacial tension, N/m

## Subscript

A: aqueous phase
cav: cavitation
cz: effective cavitation zone
m: mixture phase
*O*: organic phase
vc: lower limit
vu: upper limit

## Acronyms

AM: additive manufacturing
ANN: artificial neural network
CFD: computational fluid dynamics
DI: deionized
DSD: drop size distribution
HC: hydrodynamic cavitation
PBM: population balance model
PDF: probability density function
RO: rapeseed oil
SI: Supporting Information
SIMPLE: semi-implicit method for pressure-linked equations
SLA: stereolithography
SST: shear stress transport
URANS: unsteady Reynolds-averaged Navier–Stokes
VD: vortex-based hydrodynamic cavitation device
