# Supplementary material for: Vortex-Based Cavitation Devices for Continuous Emulsification: Influence of the Device Design, Scale-Up, and Scale-Out
Source: Ind Eng Chem Res. 2026 Apr 8;65(15):8081–101. doi: 10.1021/acs.iecr.6c00278 (PMC13107463; doi:10.1021/acs.iecr.6c00278)
Supplement: Supplementary file 1 [file ie6c00278_si_001.pdf]

Supporting Information

Vortex-based Cavitation Devices for Continuous Emulsification:
Influence of device design, scale-up and scale-out

Amol Gode[a], Kyriakos Kourousis[b] and Vivek V. Ranade[a*]
[a]Multiphase Reactors and Intensification Group
Bernal Institute, University of Limerick, Limerick V94T9PX, Ireland
[b] School of Engineering, University of Limerick, V94T9PX, Ireland
*Email: vivek.ranade@ul.ie

Sections

S1. Simulation model 2
S2. Experimental 8
S2.1 Setup photograph, schematic and 3D printed devices 8
S2.2 Emulsion stability 11
S2.3 DSD and characteristic diameters 12
S2.4 Proposed scale-out configurations 17
S3. References 18

S1. Simulation model

The CFD model equations are as follows:

Continuity:

$$\frac{\partial \rho_m}{\partial t} + \nabla \cdot \rho_m \vec{v}_m = 0 \tag{S1}$$

$\vec{v}_m$ (m/s) is the mass-averaged velocity and is defined as:

$$\vec{v}_m = \frac{\sum_{k=1}^{q} \alpha_k \rho_k \vec{v}_k}{\rho_m} \tag{S2}$$

ρ_m (kg/m^3) is mixture density given as:

$$\rho_m = \sum_{k=1}^{q} \alpha_k \rho_k \tag{S3}$$

α_k (-) is the volume fraction of phase k, and q is the total number of phases.

Momentum:

$$\frac{\partial \rho_m \vec{v}_m}{\partial t} + \nabla \cdot \rho_m \vec{v}_m \vec{v}_m = -\nabla P + \nabla \cdot [\mu_m (\nabla \vec{v}_m + \vec{v}_m^T)] + \rho_m g + F - \nabla \cdot \left[\sum_{k=1}^{q} \alpha_k \rho_k \vec{v}_{d} \right. \tag{S4}$$

$$\mu_m = \sum_{k=1}^{q} \alpha_k \mu_k \tag{S5}$$

μ_m (kg/m-s) the viscosity of mixture and F is the external body forces

$$\vec{v}_{kA} = \vec{v}_k - \vec{v}_A \tag{S6}$$

The drift velocity $\vec{\mathbf{v}}_{dr,k}$ (m/s) and slip velocity $\vec{v}_{kA}$ (m/s) is related as

$$\vec{\mathbf{v}}_{dr,k} = \vec{\mathbf{v}}_{kA} - \sum_{k=1}^{q} c_k \vec{v}_{kA} \tag{S7}$$

$\vec{\mathbf{v}}_{dr,k}$ is the drift velocity (m/s), c_k (-) mass fraction of the any phase given as:

$$c_k = \frac{\alpha_k \rho_k}{\rho_m} \tag{S8}$$

$\vec{v}_{kA}$ is slip velocity (m/s)

$$\vec{v}_{kA} = \frac{24}{\mathrm{Re}} \frac{(\rho_\mathrm{k} - \rho_\mathrm{m}) d_\mathrm{k}^2}{18 \mu_\mathrm{A} C_D} \left(g - \frac{D v_\mathrm{m}}{Dt} \right) \tag{S9}$$

$$C_D = \begin{cases} \frac{24}{Re}(1 + 0.15 Re^{0.687}) & Re \leqslant 1000 \\ 0.44 & Re > 1000 \end{cases} \text{ where Re} = \frac{\rho_m \vec{v}_{kA} d_k}{\mu_A} \tag{S10}$$

Turbulence:

$$\frac{\partial}{\partial t}(\rho k) + \frac{\partial}{\partial x_i}(\rho k u_i) = \frac{\partial}{\partial x_j}\left(\Gamma_k \frac{\partial k}{\partial x_j} \right) + G_k - Y_k + S_k, \tag{S11}$$

$$\frac{\partial}{\partial t}(\rho \omega) + \frac{\partial}{\partial \mathrm{x_i}}(\rho \omega u_\mathrm{i}) = \frac{\partial}{\partial \mathrm{x_j}}\left(\Gamma_\omega \frac{\partial \omega}{\partial \mathrm{x_j}} \right) + \mathrm{G}_\omega - Y_\omega + S_\omega, \tag{S12}$$

k: Turbulence kinetic energy (m^2/s^2), G_k and G_ω are the generation of turbulence kinetic energy k and ω, Γ_k and Γ_ω represent effective diffusivity of k and ω

Cavitation:

$$\frac{\partial}{\partial t}(\rho_\mathrm{m} f) + \nabla \cdot (\rho_\mathrm{m} \vec{v}_\mathrm{m} f) = \nabla \cdot (\Gamma \nabla f) + R_\mathrm{e} - R_\mathrm{c} \tag{S13}$$

f is the vapor mass fraction (-), Γ effective diffusion coefficient, R_e and R_c are the mass source and sink terms for the evaporation and condensation, respectively formulated as:

$$R_e = C_1 \frac{\sqrt{k}}{\sigma} \rho_v \rho_A \left[\frac{2}{3}\left(\frac{P_v - P}{\rho_A}\right)\right]^{1/2} (1 - f_v - f_g) \quad \text{(S14)}$$

$$R_c = C_2 \frac{\sqrt{k}}{\sigma} \rho_v \rho_A \left[\frac{2}{3}\left(\frac{P - P_v}{\rho_A}\right)\right]^{1/2} f_v \quad \text{(S15)}$$

ρ_v is the density of the vapor (kg/m^3), f_g represents the mass fraction of non-condensable gases, C_1 and C_2 are empirical constants having value of 0.02 and 0.01, respectively.

The PBE is written in terms of the volume fraction of drop size i as:

$$\frac{\partial \rho_m \alpha_{O,i}}{\partial t} + \nabla \cdot (\rho_m \alpha_{O,i} \vec{v}_m) = \rho_O V_i (B_i - D_i) \quad \text{(S16)}$$

where $\alpha_{O,i}$ is the oil volume fraction of representative group of drop size i. B_i and D_i are rates of birth and loss of drops of size i due to the breakage per unit volume, respectively.

$$\alpha_{O,i} = N_i V_i \quad \text{(S17)}$$

where N_i is total number of drops of size i per unit volume and calculated as:

$$N_i(t) = \int_{V_i}^{V_{i+1}} n(V,t) dV \quad \text{(S18)}$$

where, V_i is volume of representative group for drop size i.

The oil volume fraction of all the groups (α_O) is defined as:

$$\alpha_O = \sum_{i=1}^{M} \alpha_{O,i} \quad \text{(S19)}$$

The birth and loss terms were discretised as:

$$B_i = \sum_{j=i+1}^{M} p_j g(V_j) N_j \beta(V_i/V_j) \text{ and } D_i = g(V_i) N_i \quad \text{(S20)}$$

Based on the modifications suggested by Gode and Ranade for the Alopaeus et al. model for breakage frequency $g(V')$ to include breakage due to turbulent shear and cavitation, the $g(V')$ is given by:

For, $\alpha_v < \alpha_{vc}$

$$g(V') = C_2 \varepsilon^{1/3} \text{erfc}\left(\sqrt{\frac{(C_3\sigma)}{\rho_A \varepsilon^{2/3} d^{5/3}} + \frac{(C_4 \mu_O)}{\sqrt{\rho_A \rho_O} \varepsilon^{1/3} d^{4/3}}}\right)$$

(S21)

For, $\alpha_{vc} \le \alpha_v < 0.6$

$$g(V') = C_2 \varepsilon_{cav}^{1/3} \text{erfc}\left(\sqrt{\frac{(C_3\sigma)}{\rho_A \varepsilon_{cav}^{2/3} d^{5/3}} + \frac{(C_4 \mu_O)}{\sqrt{\rho_A \rho_O} \varepsilon_{cav}^{1/3} d^{4/3}}}\right)$$

here, d is drop diameter and μ_O is viscosity of the oil phase. C_2, C_3 and C_4 are model parameters. The daughter droplet distribution function $[\beta(V/V')]$ was a parabolic probability distribution function and is represented as:

$$\beta(V/V') = 0.5\left[\frac{C}{V'} + \frac{1 - C/2}{V'}\left\{24\left(\frac{V}{V'}\right)^2 - 24\left(\frac{V}{V'}\right) + 6\right\}\right] \quad \text{(S22)}$$

where, C= 1.

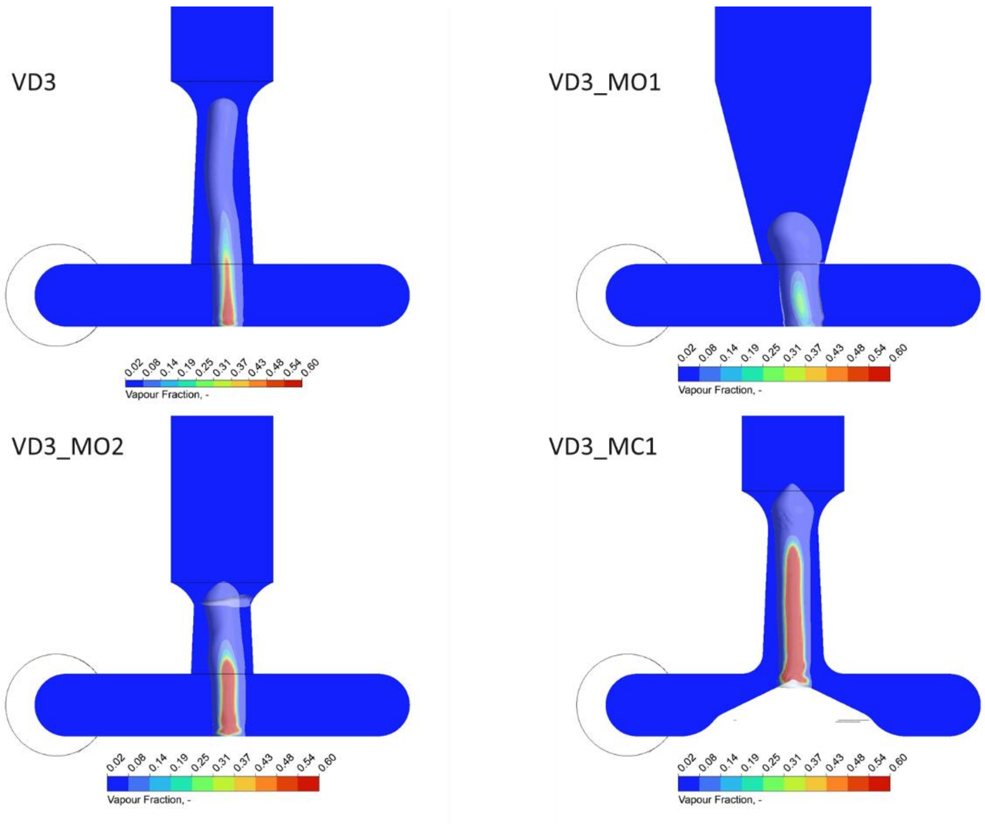

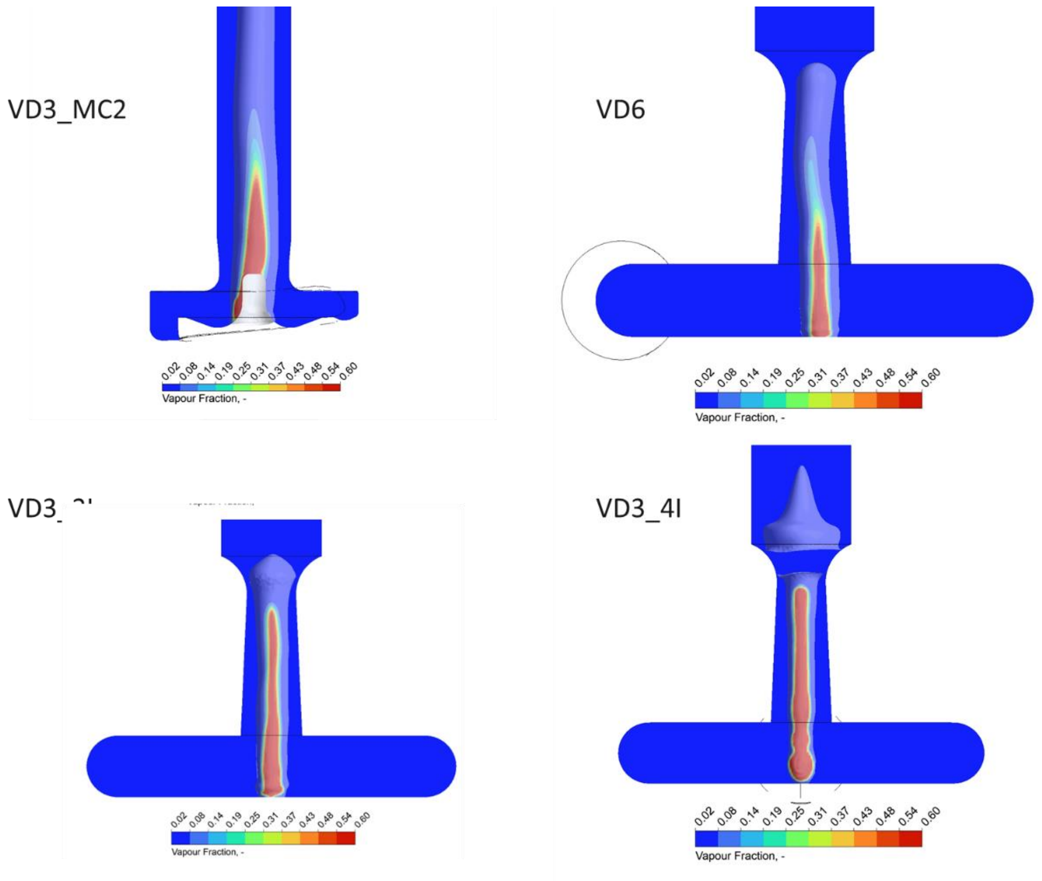

Figure S1. Contours of the effective cavitation region $0.02 \leq \alpha_v < 0.6$ for different designs

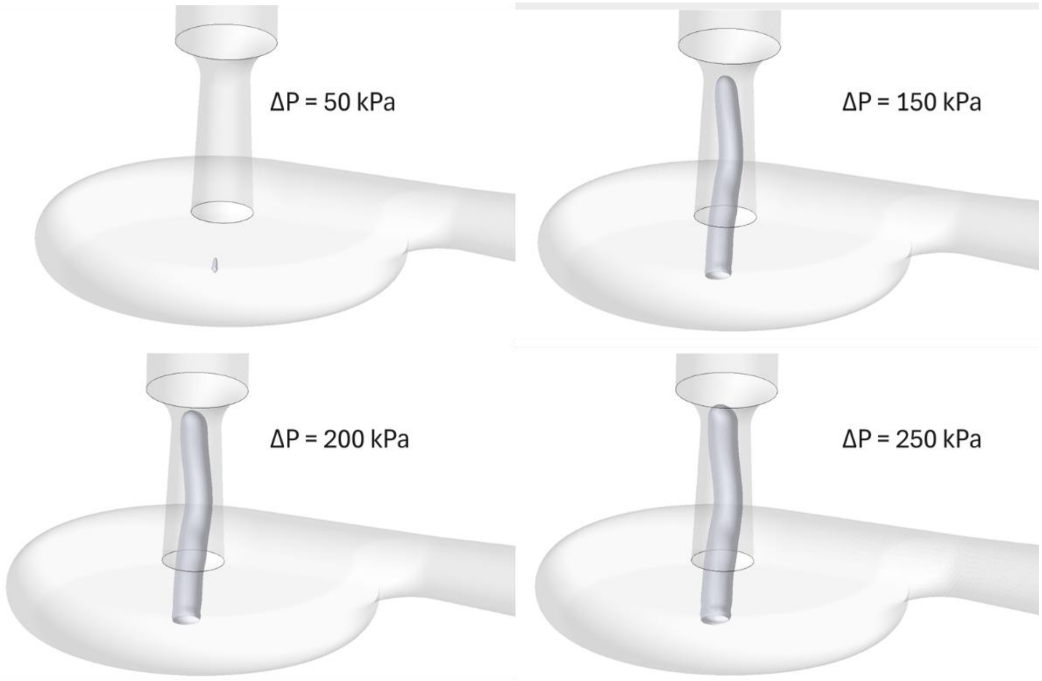

Figure S2. Variation of effective cavitation region with α_{vc}= 0.02 at ΔP = 50, 150, 200 and 250 kPA for VD3.

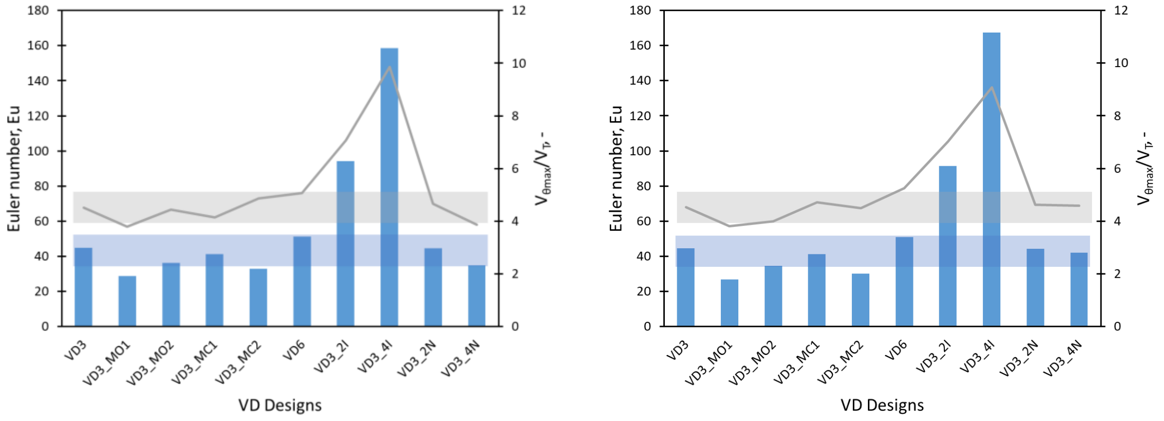

(a) (b)

Figure S3. Variation of Euler number, Eu (shown as bars) and swirl ratio ($V_{\theta max}/V_T$) (shown as lines) for different designs at (a) ΔP = 250 kPa and (b) $V_T =$ 3m/s

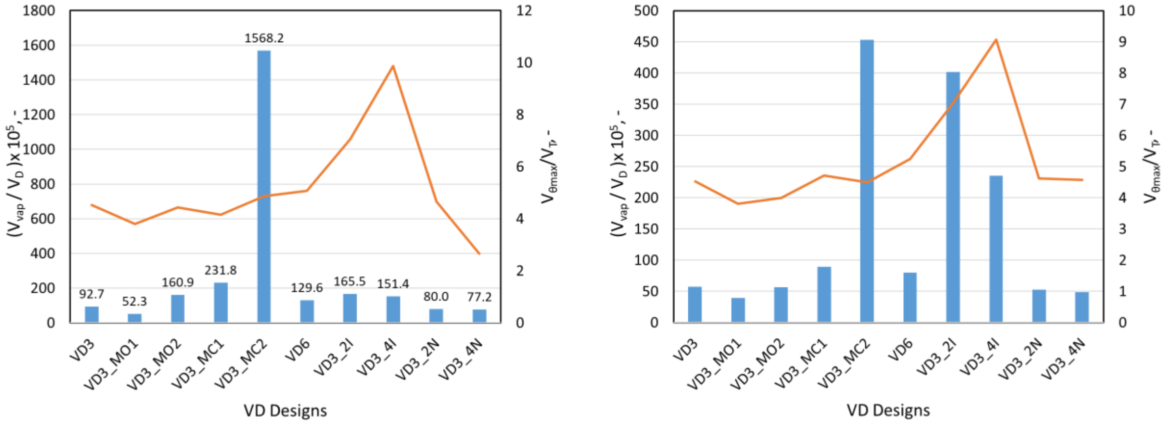

(a) (b)

Figure S4. Variation of cavitation extent, $^{V_{vap}}/_{V_D}\, x10^5$ and swirl ratio ($V_{\theta max}/V_T$) for different designs at (a) ΔP = 250 kPa and (b) $V_T =$ 3m/s

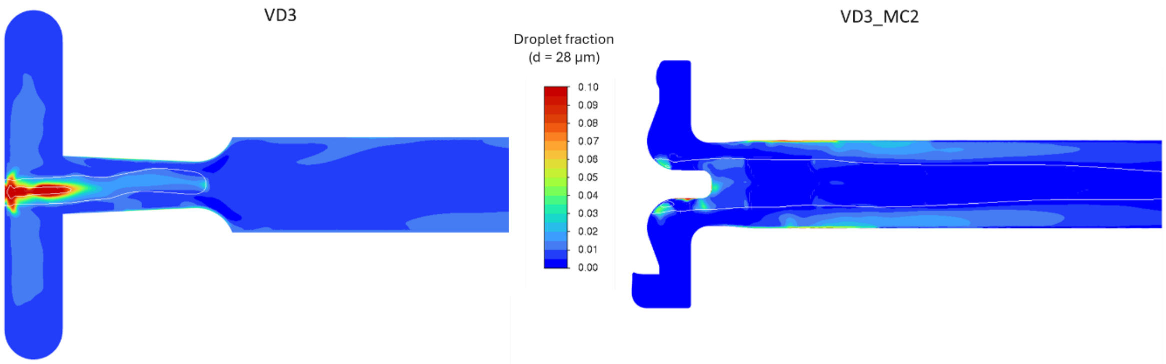

Figure S5. Comparison of location of effective cavitation region with the flow trajectories coloured by velocity magnitude.

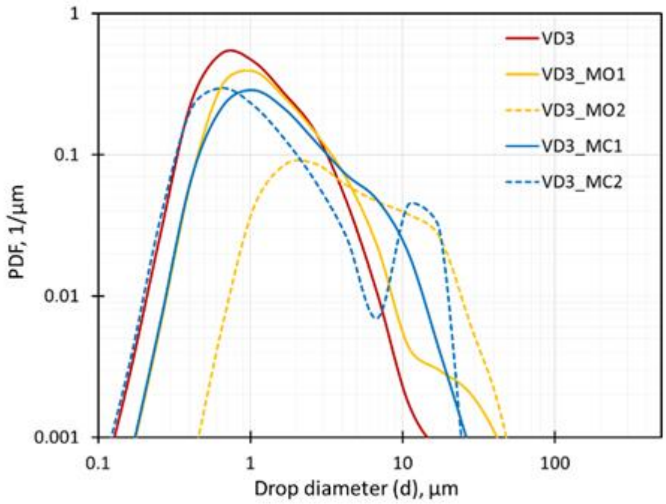

Figure S6. Comparison of simulated *DSD* for VD3, VD3_MO1, VD3_MO2, VD3_MC1 and VD3_MC2.

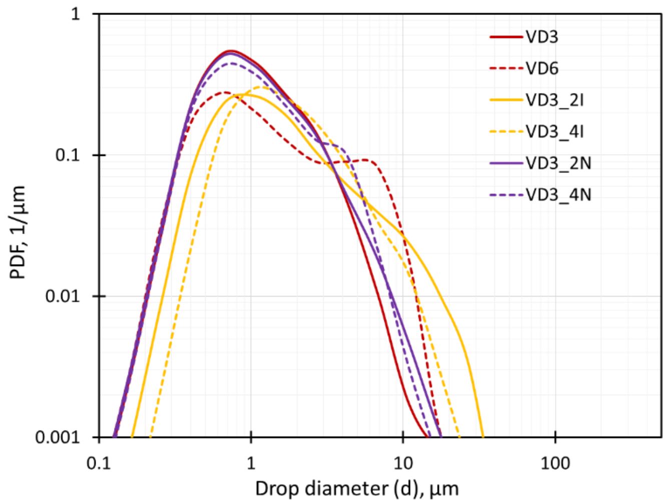

Figure S7. Comparison of simulated *DSD* for VD3, VD3_MO1, VD3_MO2, VD3_MC1 and VD3_MC2.

S2. Experimental

S2.1 Setup photograph, schematic and 3D printed devices

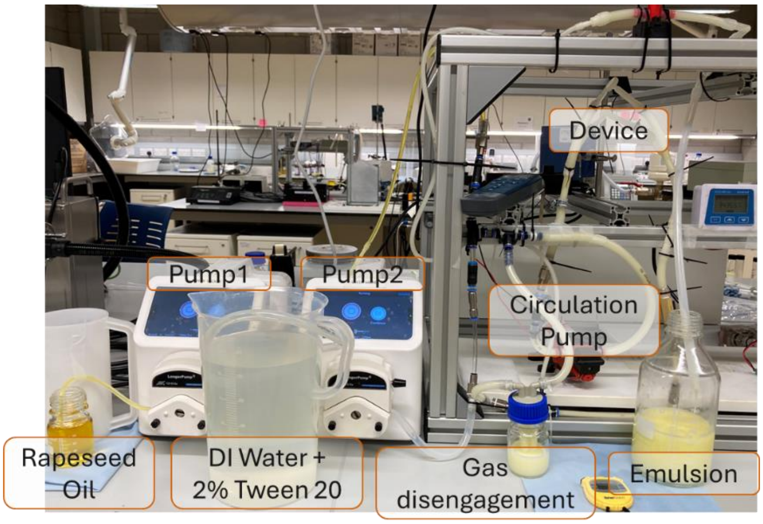

(a)

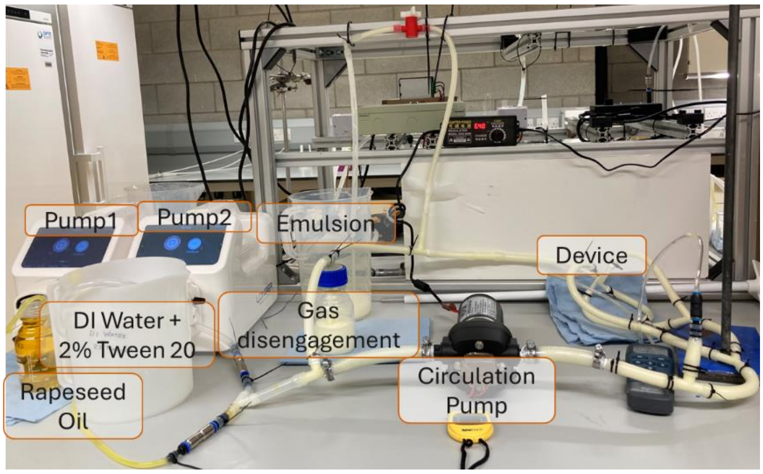

(b)

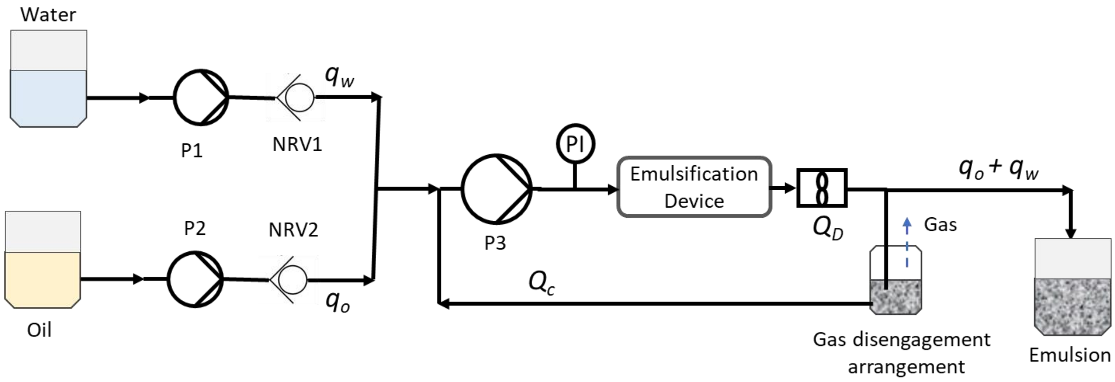

(c)

Figure S8. Experimental setup for continuous emulsion production (a) Setup for low flow rate devices (b) Setup for high flow rate devices and (c) Schematic of experimental setup used for continuous emulsion production. (reproduced from Gode and Ranade [28]).

Table S1. List of vortex-based HC devices experimentally evaluated.

**Device names**	**Throat dia. (d_T), mm**	**Chamber shape, -**	**Outlet port modifications, -**	**Number of inlets, -**	**Number of devices, -**	**Euler number (Eu), -**	**System volume (V), ml**
**VD3**	3	Cylindrical	Standard	1	1	48	120
**VD6**	6	Cylindrical	Standard	1	1	55	350
**VD3_MC1**	3	Vortex stabiliser	Standard	1	1	50	120
**VD3_MC2**	4.35	Volute shape	ER_o = 1	1	1	40	120
**VD3_2N**	3	Cylindrical	Standard	1	2	40	120
**VD3_4N**	3	Cylindrical	Standard	1	4	40	350
**VD3_2I**	3	Cylindrical	Standard	2	1	100	120
**VD3_4I**	3	Cylindrical	Standard	4	1	150	350

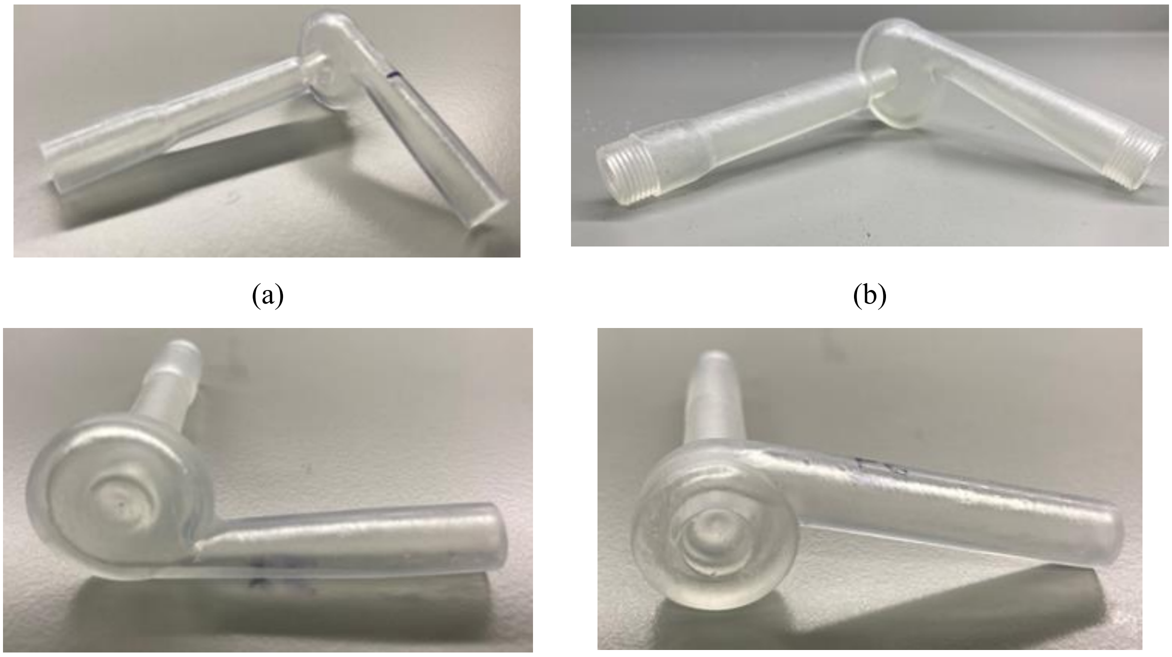

(a) (b)

(c) (d)

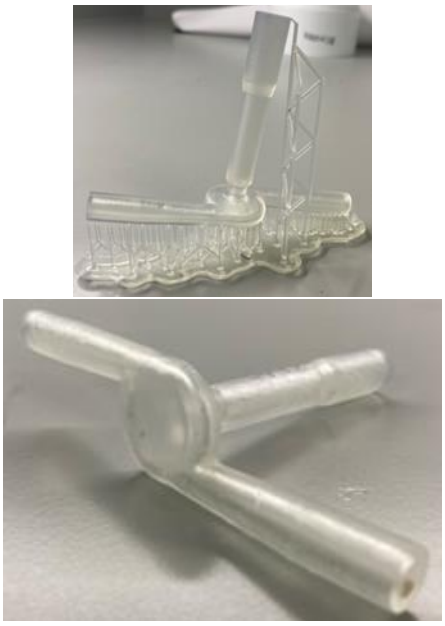

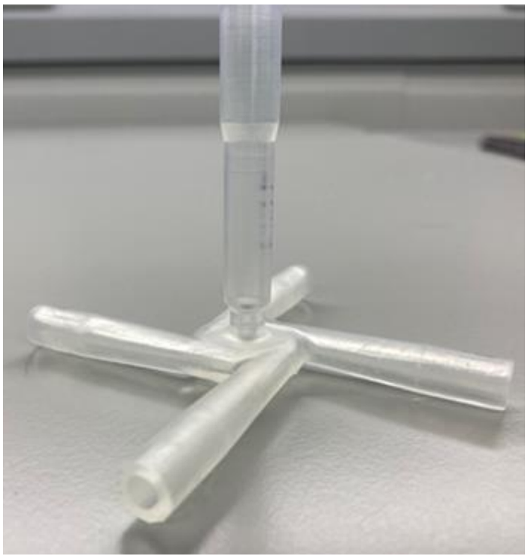

(e) (f)

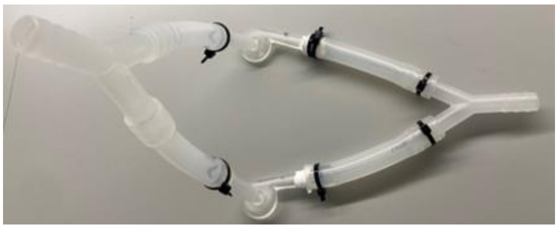

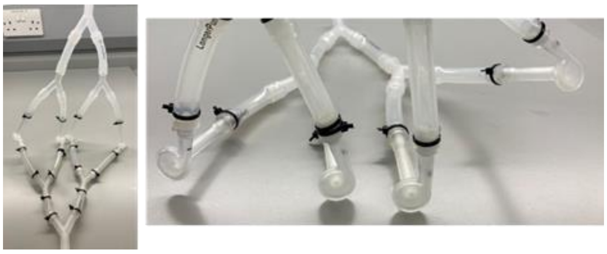

(g) (h)

Figure S9. SLA 3D printed devices used for experimental investigations

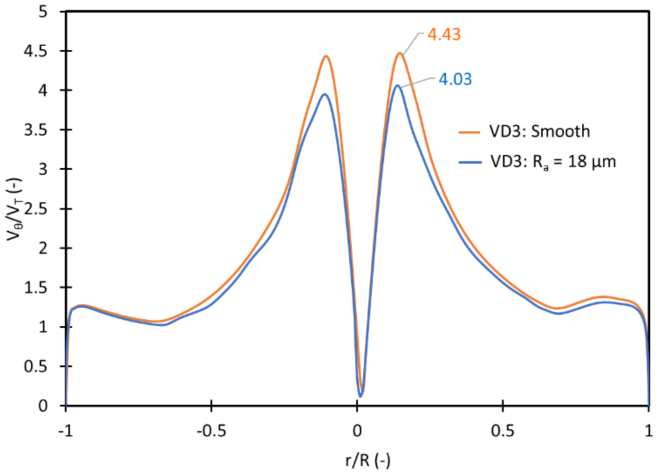

Figure S10. Influence of surface roughness on swirl ratio (V_θ/V_T) for VD3 at V_T = 3 m/s.

S2.2 Emulsion stability

The industrial applicability of continuous emulsification using VD depends on the physical and chemical stability of the final product. While the current study focuses on the evaluating VD device geometry and capacity, the stability of emulsions produced by this specific VD has been validated in our prior work [1].

Physical stability: The physical stability was evaluated through time-resolved DSD measurements in our group's previous work (Thaker and Ranade [1]). The emulsions showed negligible variation in d_{32} and DSD shape over a period of more than a month confirming that the vortex-based cavitation process produces emulsions that are highly resistant to coalescence and Ostwald ripening under the investigated conditions. This demonstrates that emulsions produced using vortex-based hydrodynamic cavitation devices are physically stable under the conditions investigated. The variation of DSD for rapeseed oil-in-water emulsion prepared at ΔP= 150 kPa and α_O= 0.2 is shown in the Figure S11 (SI) reproduced from Thaker and Ranade [1]. Regarding the Zeta potential, it is important to note that we utilized Tween 20 surfactant, which is a non-ionic surfactant that stabilizes droplets through steric hindrance provided by their bulky polyoxyethylene chains. In non-ionic systems, the Zeta potential is often close to neutral and is not the primary indicator of kinetic stability. Instead, stability is governed by the thickness and hydration of the surfactant layer at the interface. Overall, it is to be noted that because Tween 20 provides steric stabilization, long-term stability was validated via time-resolved DSD (based on previous work by Thaker and Ranade for this specific system) rather than Zeta potential.

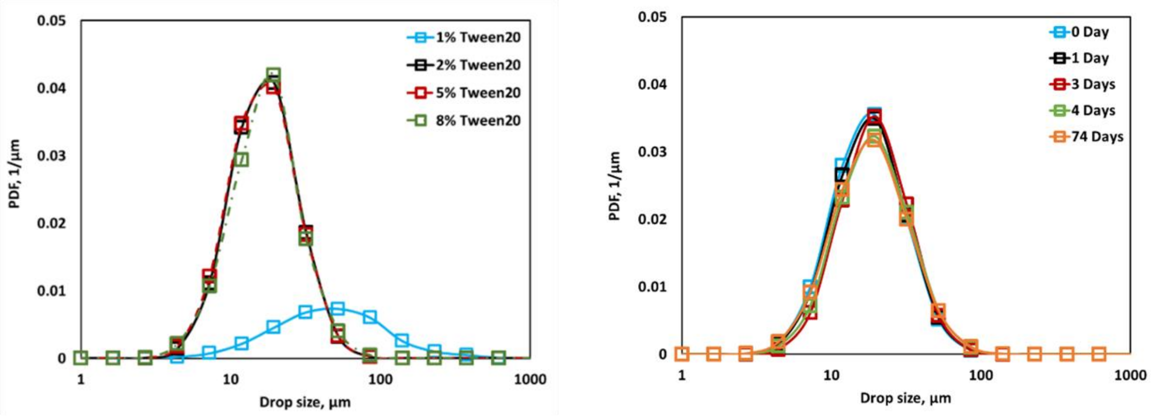

(a) (b)

Figure S11: Drop size distribution of liquid-liquid emulsion for different (a) surfactant concentrations and (b) time period (ΔP= 150 kPa, V_T= 2.36 m/s, α_O= 0.2)
(Lines are shown to indicate the overall shape of DSD) – reproduced from Thaker and Ranade [18].

Chemical stability: Hydrodynamic cavitation generates reactive species such as hydroxyl radicals which may potentially influence chemical stability of emulsions. However, several factors limit their impact on the oil phase in the present system:

- Radical generation occurs predominantly in the aqueous phase during cavity collapse. The radicals are hydrophilic and probability of reaction with oil droplets is limited to interfacial region.
- Hydroxyl radicals have extremely short lifetimes (~10^{-9} s) and correspondingly small diffusion lengths (~nm scale), which restrict their interaction with the dispersed oil phase. In this work, the oil droplets were stabilised by Tween 20, whose polyoxyethylene chains form a hydrated interfacial layer that acts as a barrier to radical transport [2].
- Hydroxyl radicals are likely to preferentially self-react rather than reacting with the oil molecules shielded by Tween 20 molecules (surfactant used in this work).
- The residence time of droplets within the cavitation-active regions is very short (~10^{-2} seconds, based on the work of Simpson and Ranade [3] using similar device VD6), limiting cumulative exposure.

Based on these considerations, significant chemical degradation of the oil phase is not expected under the operating conditions employed in this study. This is also consistent with the absence of observable changes in emulsion properties over time. This is supported by a recent studies relevant to the food industry where HC was used for the homogenization of milk and the encapsulation of sensitive bioactive compounds (e.g., omega-3 fatty acids and vitamins). These studies have shown that HC preserves the nutritional profile as the bulk temperature rise is controlled and the exposure time is minimal [4, 5]. Furthermore, Carpenter and Saharan [6] utilized FTIR analysis to study the chemical stability of mustard oil-in-water emulsions produced via intense ultrasonic cavitation. Their results proved the oil structure remained unaltered after the cavitation. Given that the residence time in our continuous VD system is orders of magnitude lower (10^0 s) than the batch ultrasonicator they evaluated (10^2 min), the chemical integrity of the oil is preserved.

S2.3 DSD and characteristic diameters

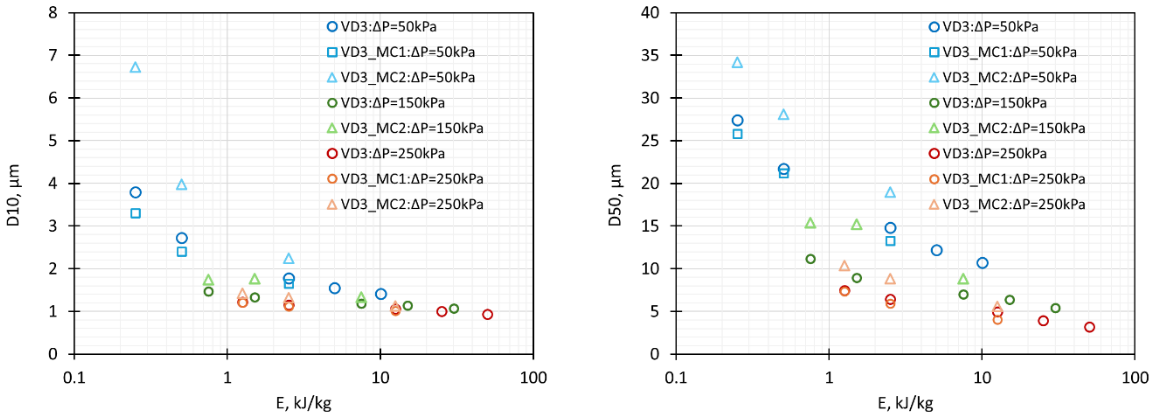

(a) (b)

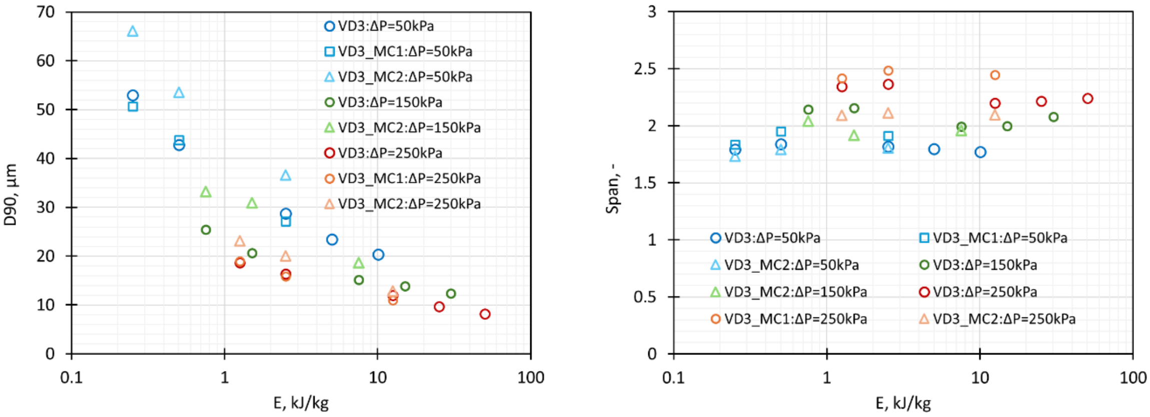

(c) (d)

Figure S12. Influence of shape: Variation of characteristic diameters ($D10$, $D50$ and $D90$) and span with E.

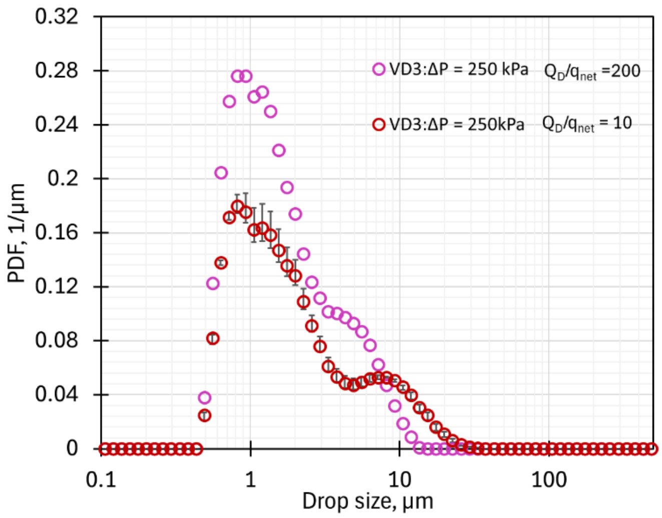

Figure S13. *DSD* at higher E ($^{Q_D}/_{q_{net}}$ = 200) value tends towards mono-modal.

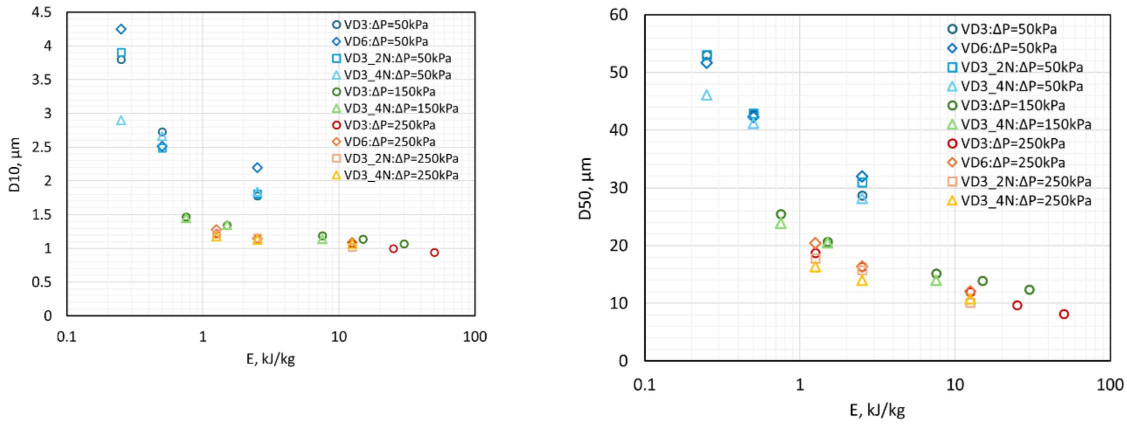

(a) (b)

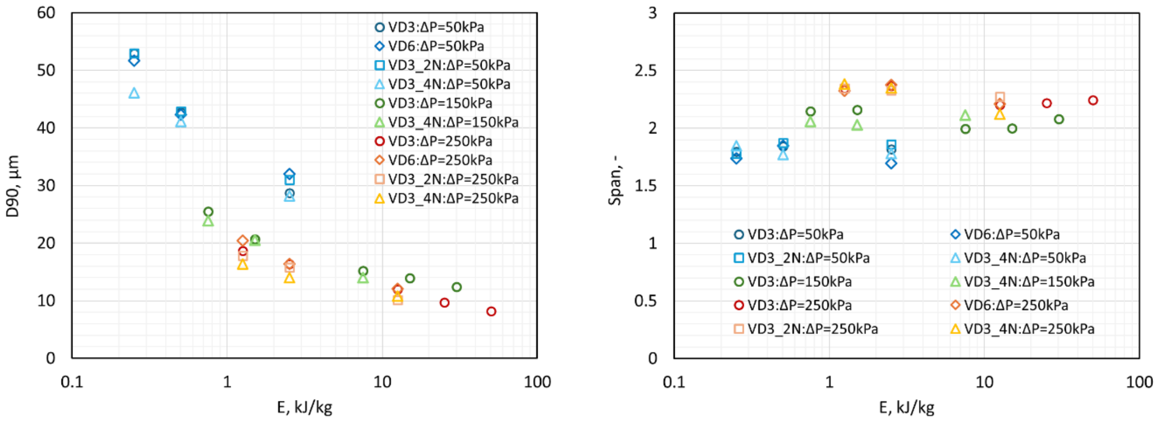

(c) (d)

Figure S14. Influence of capacity: scale-up and scale-out: Variation of characteristic diameters ($D10$, $D50$ and $D90$) and span with E.

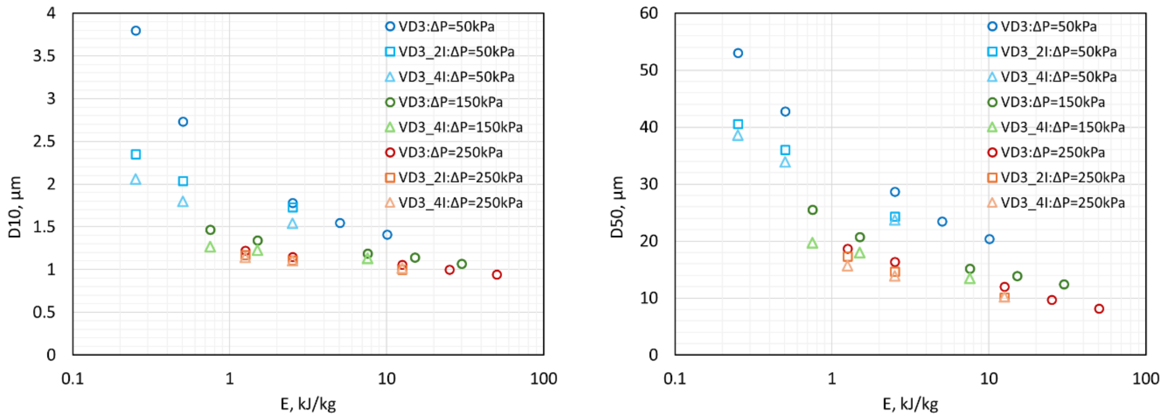

(a) (b)

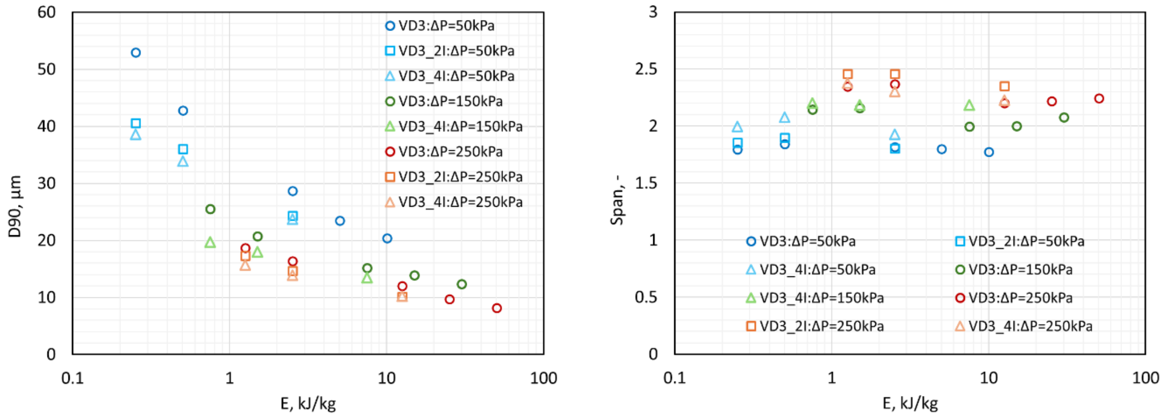

(c) (d)

Figure S15. Influence of multiple inlets: Variation of characteristic diameters ($D10$, $D50$ and $D90$) and span with E.

Table S2. Variation of characteristic diameters ($D10$, $D50$ and $D90$), span and η with E

Design	ΔP, kPa	Q_D/q_{net}, -	E, kJ/kg	$\bar{\varepsilon}$, m^2/s^3	D10, µm	D50, µm	D90, µm	d_{32}, µm	Span, -	η, %
VD3	50	5	0.25	211.7	3.8	27.4	53.0	8.8	1.8	0.48
	50	10	0.50	211.7	2.7	21.7	42.8	7.3	1.8	0.29
	50	50	2.51	211.7	1.8	14.8	28.7	5.4	1.8	0.08
	50	100	5.02	211.7	1.6	12.2	23.5	4.6	1.8	0.05
	50	200	10.04	211.7	1.4	10.7	20.4	4.2	1.8	0.03
	150	5	0.75	1037.2	1.5	11.2	25.5	4.3	2.1	0.33
	150	10	1.51	1037.2	1.3	9.0	20.7	3.7	2.2	0.19
	150	50	7.53	1037.2	1.2	7.0	15.2	3.2	2.0	0.04
	150	100	15.06	1037.2	1.1	6.4	13.9	3.0	2.0	0.02
	150	200	30.13	1037.2	1.1	5.5	12.4	2.8	2.1	0.01
	250	5	1.26	2096.4	1.2	7.5	18.7	3.3	2.3	0.26
	250	10	2.51	2096.4	1.1	6.4	16.4	3.0	2.4	0.14
	250	50	12.55	2096.4	1.1	5.0	12.0	2.7	2.2	0.03
	250	100	25.11	2096.4	1.0	3.9	9.7	2.4	2.2	0.02
	250	200	50.21	2096.4	0.9	3.2	8.2	2.2	2.2	0.01
VD3_MC1	50	5	0.25	232.8	3.3	25.8	50.7	8.4	1.8	0.50
	50	10	0.50	232.8	2.4	21.2	43.8	7.1	2.0	0.30
	50	50	2.51	232.8	1.7	13.3	27.1	4.9	1.9	0.09
	250	5	1.26	2222.4	1.2	7.4	19.0	3.3	2.4	0.26
	250	10	2.51	2222.4	1.1	5.9	15.9	2.9	2.5	0.14
	250	50	12.55	2222.4	1.0	4.1	11.0	2.4	2.4	0.03
VD3_MC2	50	5	0.25	1498.0	6.7	34.2	66.1	10.9	1.7	0.39
	50	10	0.50	1498.0	4.0	28.1	53.6	8.9	1.8	0.24
	50	50	2.51	1498.0	2.3	19.0	36.6	6.6	1.8	0.06
	150	5	0.75	7783.6	1.7	15.4	33.2	5.3	2.0	0.26
	150	10	1.51	7783.6	1.8	15.2	30.9	5.4	1.9	0.13
	150	50	7.53	7783.6	1.3	8.9	18.7	3.7	2.0	0.04
	250	5	1.26	15888.2	1.4	10.4	23.2	4.1	2.1	0.21
	250	10	2.51	15888.2	1.3	8.8	20.0	3.7	2.1	0.11
	250	50	12.55	15888.2	1.1	5.6	12.9	2.9	2.1	0.03
VD6	50	5	0.25	127.2	4.3	27.3	51.7	9.2	1.7	0.46
	50	10	0.50	127.2	2.5	21.6	42.3	7.2	1.8	0.29
	50	50	2.51	127.2	2.2	17.6	32.0	6.4	1.7	0.07
	250	5	1.26	1331.7	1.3	8.2	20.4	3.5	2.3	0.24
	250	10	2.51	1331.7	1.1	6.4	16.4	3.0	2.4	0.14
	250	50	12.55	1331.7	1.1	5.0	12.1	2.7	2.2	0.03
VD3_2N	50	5	0.25	226.3	3.9	27.6	53.0	8.9	1.8	0.47

	50	10	0.50	226.3	2.5	21.6	42.9	7.2	1.9	0.29
	50	50	2.51	226.3	1.8	15.7	31.0	5.5	1.9	0.08
	250	5	1.26	2160.9	1.2	7.1	17.8	3.2	2.3	0.26
	250	10	2.51	2160.9	1.2	6.3	15.8	3.0	2.3	0.14
	250	50	12.55	2160.9	1.0	4.0	10.1	2.4	2.3	0.03
	50	5	0.25	226.3	2.9	23.4	46.1	7.9	1.8	0.53
	50	10	0.50	226.3	2.7	21.6	41.2	7.4	1.8	0.29
	50	50	2.51	226.3	1.8	14.8	28.2	5.5	1.8	0.08
	150	5	0.75	994.0	1.5	10.9	23.9	4.2	2.1	0.34
VD3_4N	150	10	1.51	994.0	1.4	9.4	20.5	3.8	2.0	0.18
	150	50	7.53	994.0	1.1	6.1	14.0	3.0	2.1	0.05
	250	5	1.26	2160.9	1.2	6.4	16.4	3.1	2.4	0.27
	250	10	2.51	2160.9	1.1	5.5	14.0	2.9	2.3	0.15
	250	50	12.55	2160.9	1.1	4.6	10.8	2.6	2.1	0.03
	50	5	0.25	218.0	2.4	20.6	40.6	7.0	1.9	0.60
	50	10	0.50	218.0	2.0	17.9	36.0	6.2	1.9	0.34
	50	50	2.51	218.0	1.7	12.5	24.3	4.9	1.8	0.09
	250	5	1.26	2225.4	1.2	6.6	17.3	3.1	2.5	0.27
	250	10	2.51	2225.4	1.1	5.5	14.7	2.8	2.5	0.15
VD3_2I	250	50	12.55	2225.4	1.0	3.9	10.1	2.4	2.4	0.04
	241	5	1.21	2099.8	1.2	7.2	18.7	3.2	2.4	0.27
	241	10	2.42	2099.8	1.1	5.8	15.1	2.9	2.4	0.15
	241	50	12.10	2099.8	1.0	4.3	10.9	2.5	2.3	0.04
	241	100	24.20	2099.8	1.0	4.2	9.1	2.5	1.9	0.02
	241	200	48.41	2099.8	0.9	3.4	8.2	2.2	2.2	0.01
	50	5	0.25	355.2	2.1	18.3	38.6	6.2	2.0	0.68
	50	10	0.50	355.2	1.8	15.5	33.9	5.4	2.1	0.39
	50	50	2.51	355.2	1.5	11.5	23.7	4.5	1.9	0.09
	150	5	0.75	1415.6	1.3	8.4	19.7	3.5	2.2	0.40
	150	10	1.51	1415.6	1.2	7.7	18.0	3.3	2.2	0.21
	150	50	7.53	1415.6	1.1	5.7	13.5	2.9	2.2	0.05
VD3_4I	250	5	1.26	2911.9	1.1	6.1	15.7	3.0	2.4	0.28
	250	10	2.51	2911.9	1.1	5.6	13.9	2.8	2.3	0.15
	250	50	12.55	2911.9	1.0	4.1	10.2	2.5	2.2	0.03
	203	5	1.02	2095.7	1.2	7.9	18.7	3.4	2.2	0.31
	203	10	2.03	2095.7	1.2	7.1	16.6	3.2	2.2	0.16
	203	50	10.17	2095.7	1.1	5.2	12.1	2.7	2.1	0.04
	203	100	20.35	2095.7	1.0	4.4	10.3	2.5	2.1	0.02
	203	200	40.70	2095.7	1.0	3.8	9.3	2.3	2.2	0.01

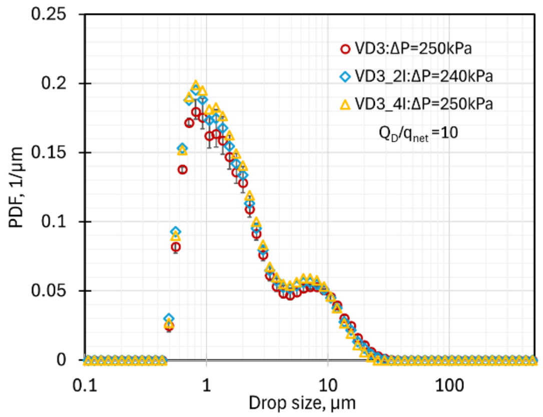

(a)

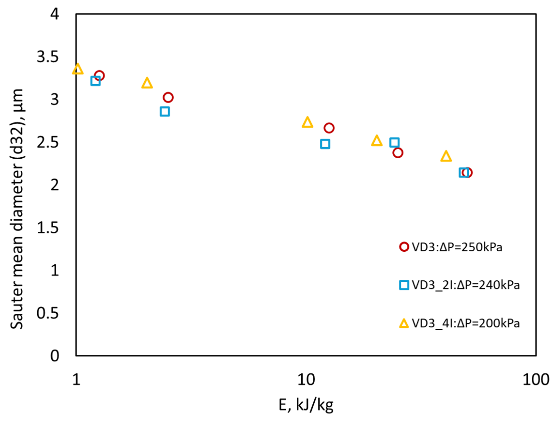

(b)

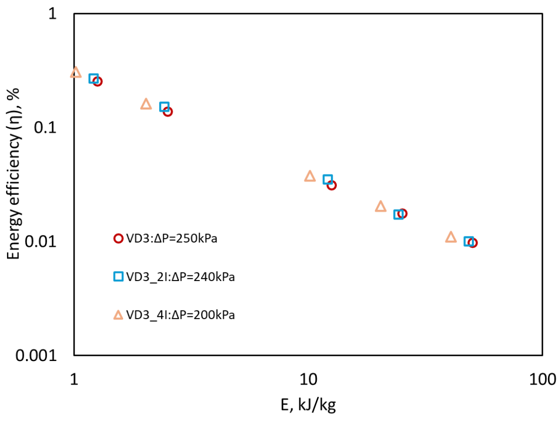

(c)

Figure S16. Variation in *DSD*, d_{32} and η at same average turbulent energy dissipation rates ($\bar{\varepsilon}$) for standard device (VD3) and multi-inlet devices (VD3_2I and VD3_4I).

S2.4 Proposed scale-out configurations

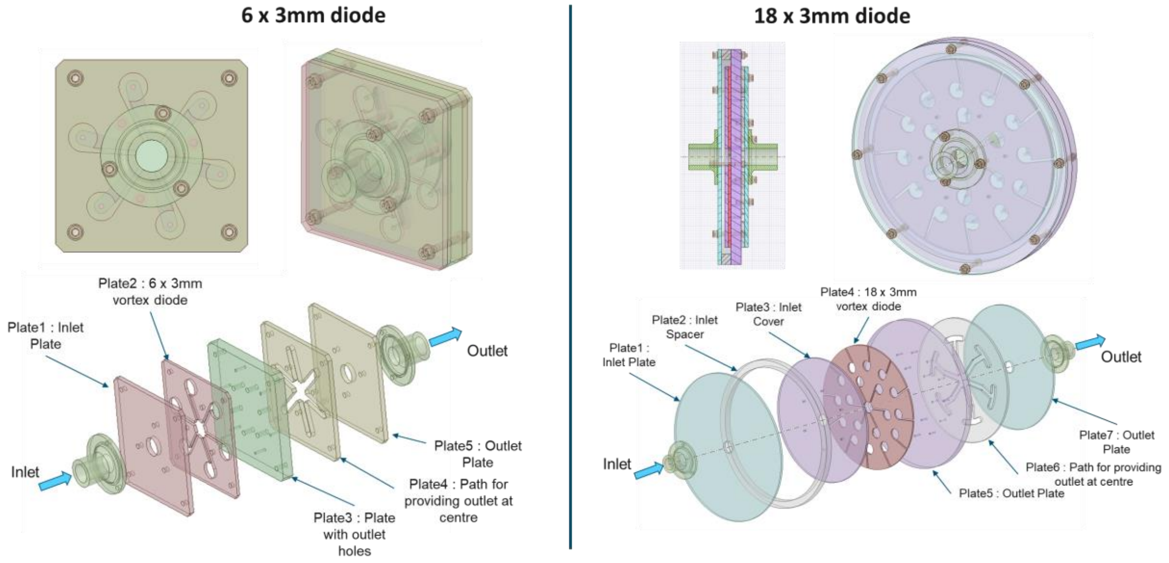

(a)

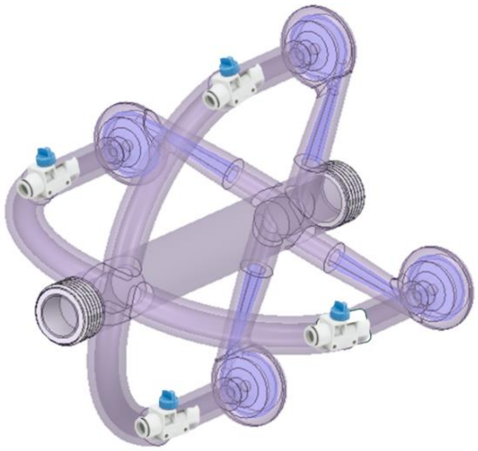

(b)

Figure S17. Scale-out configurations to change the number of active devices on demand.

S3. References

[1] A. H. Thaker and V. V. Ranade, "Drop breakage in a single-pass through vortex-based cavitation device: Experiments and modeling," *AIChE Journal,* vol. 69, no. 1, p. e17512, 2023/01/01 2023, doi: 10.1002/aic.17512.

[2] M. Ganguly, D. Debraj, N. Mazumder, J. Carpenter, S. Manickam, and A. B. Pandit, "Impact of Ultrasonication on the Oxidative Stability of Oil-in-Water Nanoemulsions: Investigations into Kinetics and Strategies to Control Lipid Oxidation," *Industrial & Engineering Chemistry Research,* vol. 63, no. 23, pp. 10212-10225, 2024/06/12 2024, doi: 10.1021/acs.iecr.4c00506.

[3] A. Simpson and V. V. Ranade, "Flow characteristics of vortex based cavitation devices," *AIChE Journal,* vol. 65, no. 9, 2019, doi: 10.1002/aic.16675.

[4] D. Panda, V. K. Saharan, and S. Manickam, "Controlled Hydrodynamic Cavitation: A Review of Recent Advances and Perspectives for Greener Processing," *Processes*, vol. 8, no. 2, p. 220doi: 10.3390/pr8020220.

[5] S. S. Arya, P. R. More, M. R. Ladole, K. Pegu, and A. B. Pandit, "Non-thermal, energy efficient hydrodynamic cavitation for food processing, process intensification and extraction of natural bioactives: A review," (in eng), *Ultrason Sonochem,* vol. 98, p. 106504, Aug 2023, doi: 10.1016/j.ultsonch.2023.106504.

[6] J. Carpenter and V. K. Saharan, "Ultrasonic assisted formation and stability of mustard oil in water nanoemulsion: Effect of process parameters and their optimization," *Ultrasonics Sonochemistry,* vol. 35, pp. 422-430, 2017/03/01/ 2017, doi: 10.1016/j.ultsonch.2016.10.021.
